# C(sp^3^)-Arylation by Conformationally Accelerated Intramolecular Nucleophilic Aromatic Substitution (S_N_Ar)

**DOI:** 10.1021/acs.accounts.2c00184

**Published:** 2022-05-27

**Authors:** Steven M. Wales, Rakesh K. Saunthwal, Jonathan Clayden

**Affiliations:** School of Chemistry, University of Bristol, Cantock’s Close, Bristol BS8 1TS, U.K.

## Abstract

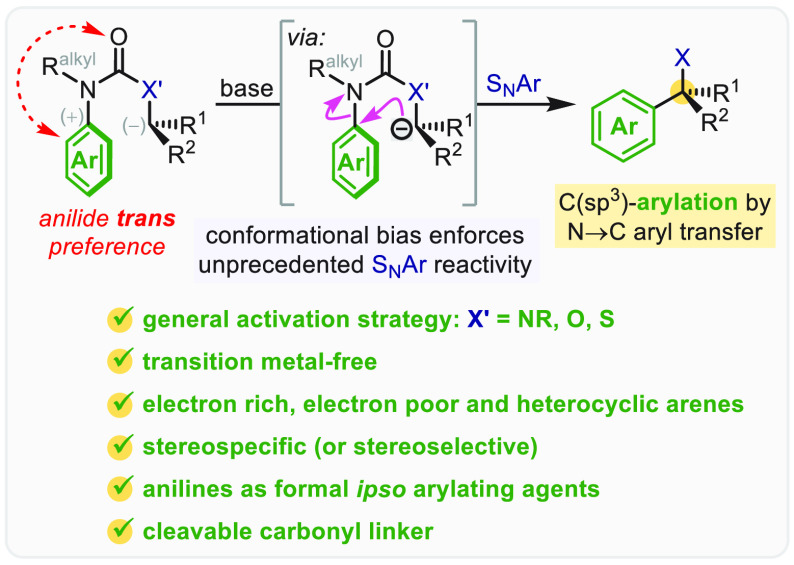

The asymmetric synthesis of heavily substituted benzylic stereogenic centers, prevalent in natural products, therapeutics, agrochemicals, and catalysts, is an ongoing challenge. In this Account, we outline our contribution to this endeavor, describing our discovery of a series of new reactions that not only have synthetic applicability but also present significant mechanistic intrigue. The story originated from our longstanding interest in the stereochemistry and reactivity of functionalized organolithiums. While investigating the lithiation chemistry of ureas (a “Cinderella” sister of the more established amides and carbamates), we noted an unexpected Truce–Smiles (T-S) rearrangement involving the 1,4-N → C transposition of a urea *N*′-aryl group to the α-carbanion of an adjacent *N*-benzyl group. Despite this reaction formally constituting an S_N_Ar substitution, we found it to be remarkably tolerant of the electronic properties of the migrating aryl substituent and the degree of substitution at the carbanion. Moreover, in contrast to classical S_N_Ar reactions, the rearrangement was sufficiently rapid that it took place under conditions compatible with configurational stability in an organolithium intermediate, enabling enantiospecific arylation at benzylic stereogenic centers. Experimental and computational studies confirmed a low kinetic barrier to the aryl migration arising from the strong preference for a *trans* arrangement of the urea *N*′-aryl and carbonyl groups, populating a reactive conformer in which spatial proximity was enforced between the carbanion and *N*′-aryl group, hugely accelerating *ipso*-substitution.

This discovery led us to uncover a whole series of conformationally accelerated intramolecular N → C aryl transfers using different anilide-based functional groups, including a diverse range of urea, carbamate, and thiocarbamate-substituted anions. Products included enantioenriched α-tertiary amines (including α-arylated N-heterocycles) and alcohols, as well as rare α-tertiary thiols. Synthetically challenging diarylated centers with differentiated aryl groups featured heavily in all product sets. The absolute enantiospecificity (retention versus inversion) of the reaction was dependent on the heteroatom α to the lithiation site: the origin of this stereodivergence was probed both experimentally and computationally. Asymmetric variants of the rearrangement were realized by enantioselective deprotonation, and connective strategies were developed in which an intermolecular C–C bond-forming event preceded the anionic rearrangement. Substrates where the *N*′-nucleofuge (at the aryl *ipso* position) was tethered to the migrating arene allowed us to use the rearrangement as a ring expansion method to generate 8- to 12-membered medium-ring N-heterocycles from very simple precursors. Stabilized carbon nucleophiles such as alkali metal enolates also readily promoted intramolecular N → C aryl transfer in *N*′-arylureas, opening up access to biologically relevant hydantoins, and enabling a “chiral memory” approach for the (hetero)arylation of chiral α-amino acids with programmable retention or inversion of configuration. Collectively, our studies of electronically versatile T-S rearrangements in anilide-based systems have culminated in a practical and general strategy for transition metal-free C(sp^3^)-arylation. More broadly, our results highlight the power of conformational activation to achieve unprecedented reactivity in the construction of challenging C–C bonds.

## Key References

- LeonardD. J.; WardJ. W.; ClaydenJ.Asymmetric α-Arylation of Amino Acids. Nature2018, 562, 105–1093028310310.1038/s41586-018-0553-9.^1^*Arene-tethered enolates derived from chiral α-amino acids undergo N′ → C_α_ aryl transfer with both electron-rich and -poor arenes. The process is formally enantiospecific and can be engineered for either retention or inversion of configuration.*
- ClaydenJ.; DufourJ.; GraingerD. M.; HelliwellM.Substituted Diarylmethylamines by Stereospecific Intramolecular Electrophilic Arylation of Lithiated Ureas. J. Am. Chem. Soc.2007, 129, 7488–74891752118910.1021/ja071523a.^2^*The first report of urea tethers in conformationally accelerated S*_*N*_*Ar reactions of carbon(sp*^*3*^*) nucleophiles, including enantiospecific C-arylation*.

## 1 Introduction

**Figure 1:**
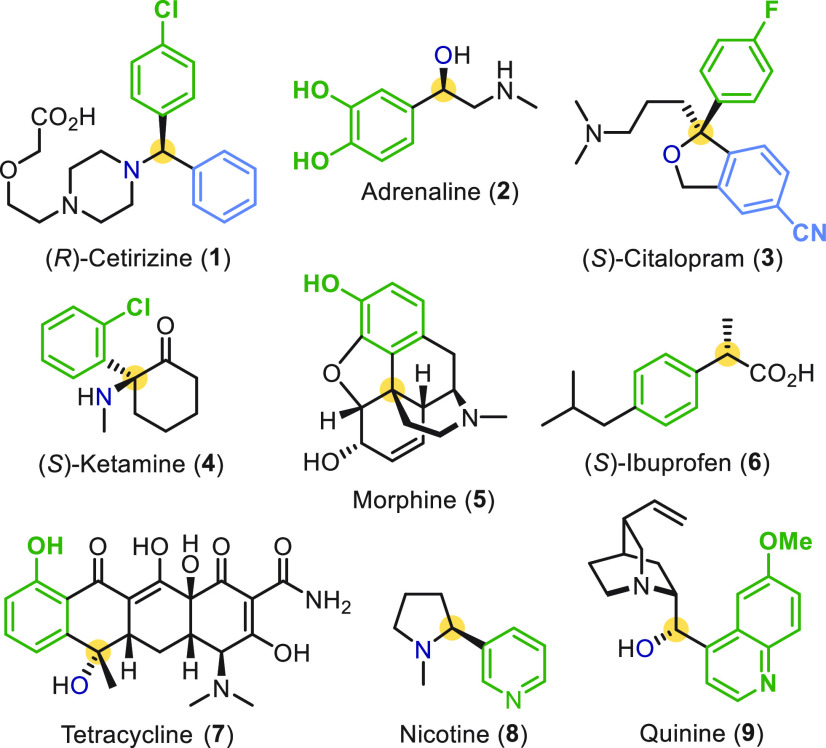
Valuable compounds containing a benzylic stereocenter.

Asymmetric carbon–carbon bond formation is a central theme in organic chemistry, with the stereoselective introduction of aromatic rings to carbon frameworks of particular importance due to the prevalence of benzylic stereocenters in functional organic molecules (e.g., **1**–**9**, Figure 1).

**Scheme 1:**
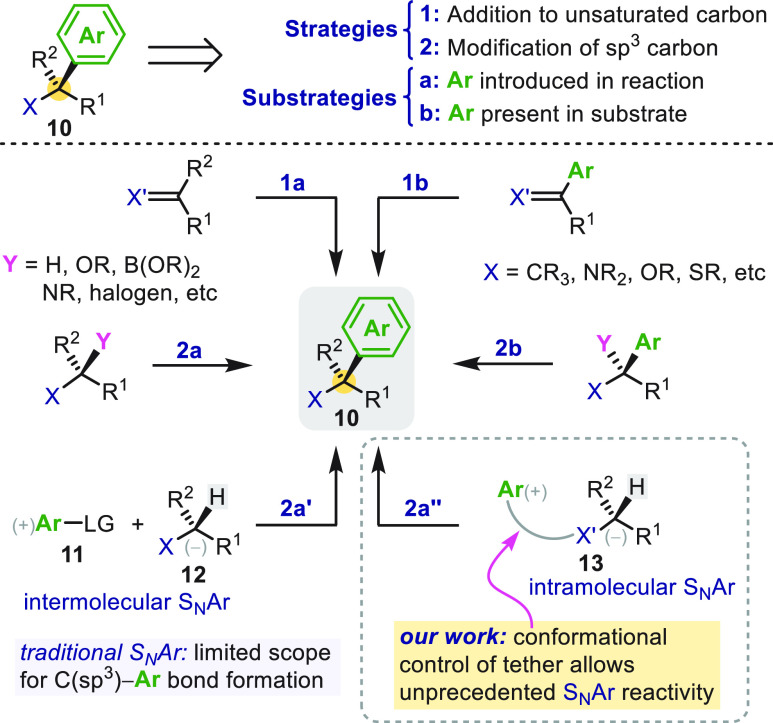
S_N_Ar Reaction and the Construction of Benzylic Stereocenters

Benzylic stereocenters are typically built either by asymmetric polar or radical additions to unsaturated carbon-based functionality (Scheme 1, strategy 1a/b) or by modification at an existing tertiary or quaternary (sp^3^)-carbon (strategy 2a/b). In these approaches, the aryl ring may be introduced during the reaction (substrategy a) or alternatively may already present as a substituent (substrategy b). Although asymmetric methods within each of these conceptual frameworks have been developed,^3−13^ the construction of fully substituted benzylic stereocenters with control of absolute stereochemistry remains a general challenge, especially in acyclic systems.^14^

Arylation of carbon-(sp^3^) (pro)nucleophiles is most commonly achieved using transition metal-catalyzed reactions of aryl halides.^15,16^ But from a conceptual standpoint, a nucleophilic aromatic substitution (S_N_Ar) reaction between a carbon (pro)nucleophile **12** and an aryl electrophile **11** also appears a viable strategy for the direct arylation of acidic C(sp^3^)–H bonds (Scheme 1, strategy 2a′). However, even for intramolecular variants that use tethered substrates **13** (strategy 2a″), the so-called Truce–Smiles (T-S) rearrangement,^17−19^ these reactions have traditionally been confined to typically S_N_Ar-reactive substrates in which the aryl electrophile carries anion stabilizing groups.^20−26^

In this Account, we outline the discovery and development of a new class of transition metal-free, intramolecular C(sp^3^)-arylations that, although formally S_N_Ar reactions, offer remarkably broad scope and utility for the preparation of quaternary benzylic stereocenters, including full control of absolute configuration. The electronic versatility of these reactions, which do not require electron-deficient aryl electrophiles, arises from substrate activation by an often underappreciated but crucial determiner of molecular reactivity: *conformational control* (Scheme 1, strategy 2a″).

**Scheme 2:**
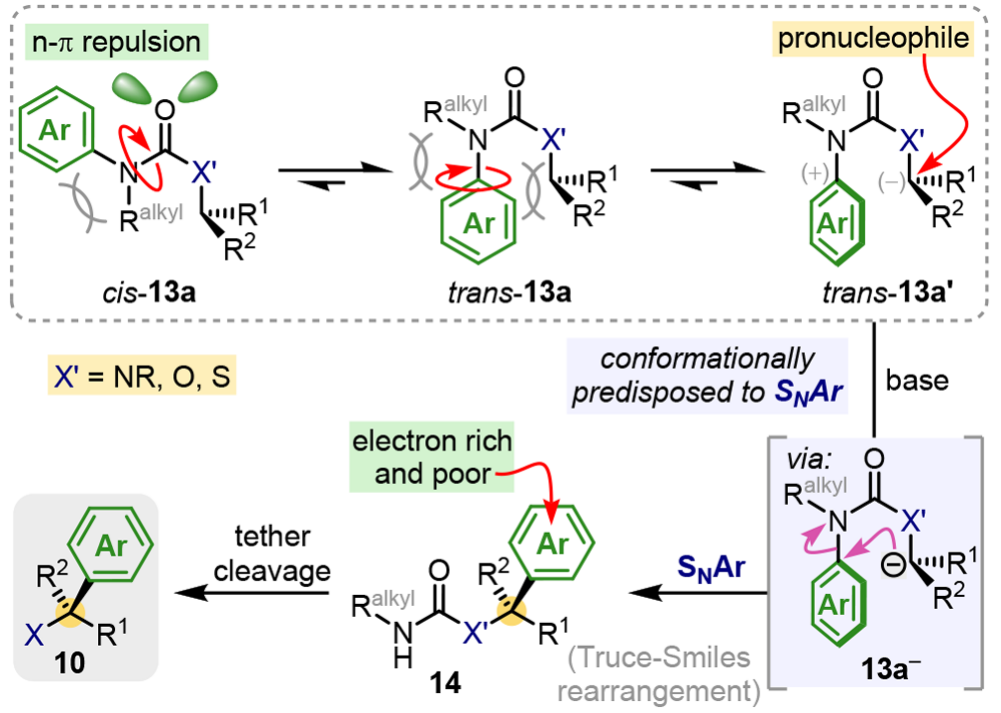
Conformationally Accelerated S_N_Ar

Conformational acceleration in intramolecular S_N_Ar reactions of **13** requires an appropriate tether to enforce the spatial proximity of the carbon nucleophile and the aryl electrophile (Scheme 1). We have found that *N*-alkyl anilides and their congeners are universally effective as tethers, owing to the *trans* conformational preference of their carbonyl and aromatic (or other π-electron-rich) groups,^27−29^ which organizes them into a conformation primed for a T-S-like intramolecular S_N_Ar reaction.^30^ This concept is illustrated in Scheme 2 for the general substrate **13a**, where a carbon pronucleophile is tethered to an arene via a fully substituted anilide nitrogen atom. The conformational preference of **13a** is dictated by unfavorable steric and electronic interactions in *cis*-**13a**, enforcing a strong bias for the *trans* conformer. Steric interactions are further relieved by rotation about the N–aryl bond in *trans*-**13a**, disrupting conjugation as the aryl ring twists perpendicular to the carbonyl plane. The “reactive” conformer *trans*-**13a′** is thus favored, with the arene π-system directly available for attack by the adjacent carbon nucleophile.

The conformational predisposition of **13a**^**–**^ lowers the activation energy for S_N_Ar sufficiently to allow N → C aryl transfer by mechanisms that apparently bypass discrete Meisenheimer intermediates and, instead, follow partially concerted trajectories.^31−34^ As a result, these reactions break free of the normal requirement for arene activation by electron-withdrawing groups. S_N_Ar reactions of **13a**^**–**^ involving highly nucleophilic carbanions are rapid enough to enable benzylic and allylic tertiary C–H bonds to be deprotonated and arylated enantiospecifically, even with electron-rich arenes.

Several additional features contribute to the general utility of these “nonclassical” T-S rearrangements: (1) The electrophilic arylating agents are formally inexpensive and readily available anilines, rather than halogenated arenes. (2) The substrates **13a** are readily prepared by classical methods that make use of commercially available isocyanate or carbamoyl chloride derivatives. (3) The remains of the tether is readily cleaved after rearrangement, providing an overall “traceless” method. (4) The conformational preferences described extend across ureas, carbamates, and thiocarbamates, allowing arylation α to nitrogen, oxygen, or sulfur (“X” in Scheme 2).

## 2 N → C Aryl Migration in Ureas: Synthesis of α-Tertiary Amines

### 2.1 Reaction Discovery and Mechanistic Studies

**Scheme 3:**
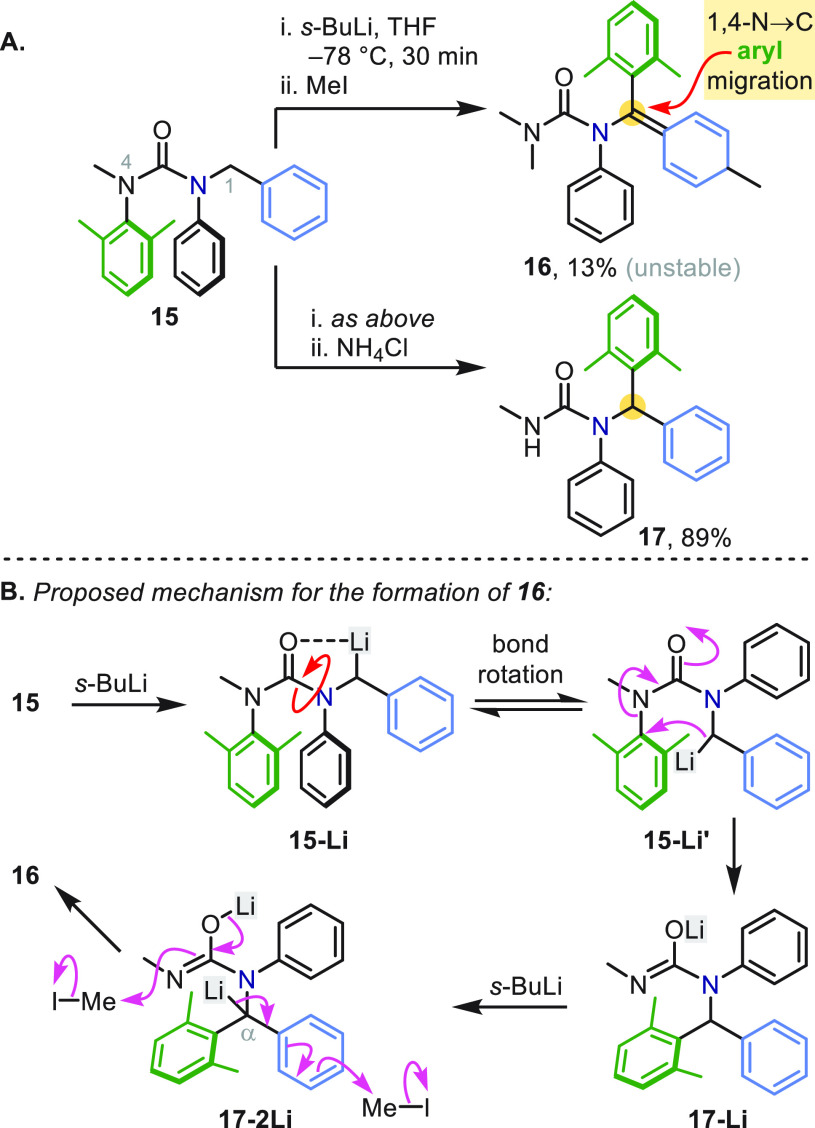
Discovery of N → C Aryl Migration

In connection with our work on *N*,*N*′-diarylureas as conformational controllers in molecular communication devices,^35^ we had cause to investigate the functionalization of urea **15** by lithiation chemistry (Scheme 3a).^36,37^ To determine the preferred site of lithiation, **15** was treated with excess *s*-BuLi, aiming to methylate with MeI. Remarkably, the dearomatized derivative **16** was the major (though unstable) product.^2^ We were immediately intrigued that **16** had apparently been formed via a sequence of events that involved intramolecular nucleophilic attack of benzyllithium **15-Li′** (Scheme 3b) on the adjacent 2,6-dimethylphenyl ring (colored green), resulting in a 1,4-N → C aryl transfer. In support of this proposal, quenching the reaction with NH_4_Cl instead of MeI gave a T-S rearrangement product **17** in excellent yield (Scheme 3a), presumably via *N*- and α-protonation of **17-2Li**.^2^ Considered alongside conventional S_N_Ar reactivity, the fact that the arene in **15** was electron-rich, sterically encumbered, and prone to migration even at low temperature made the discovery of this T-S rearrangement all the more remarkable.^38^

**Scheme 4:**
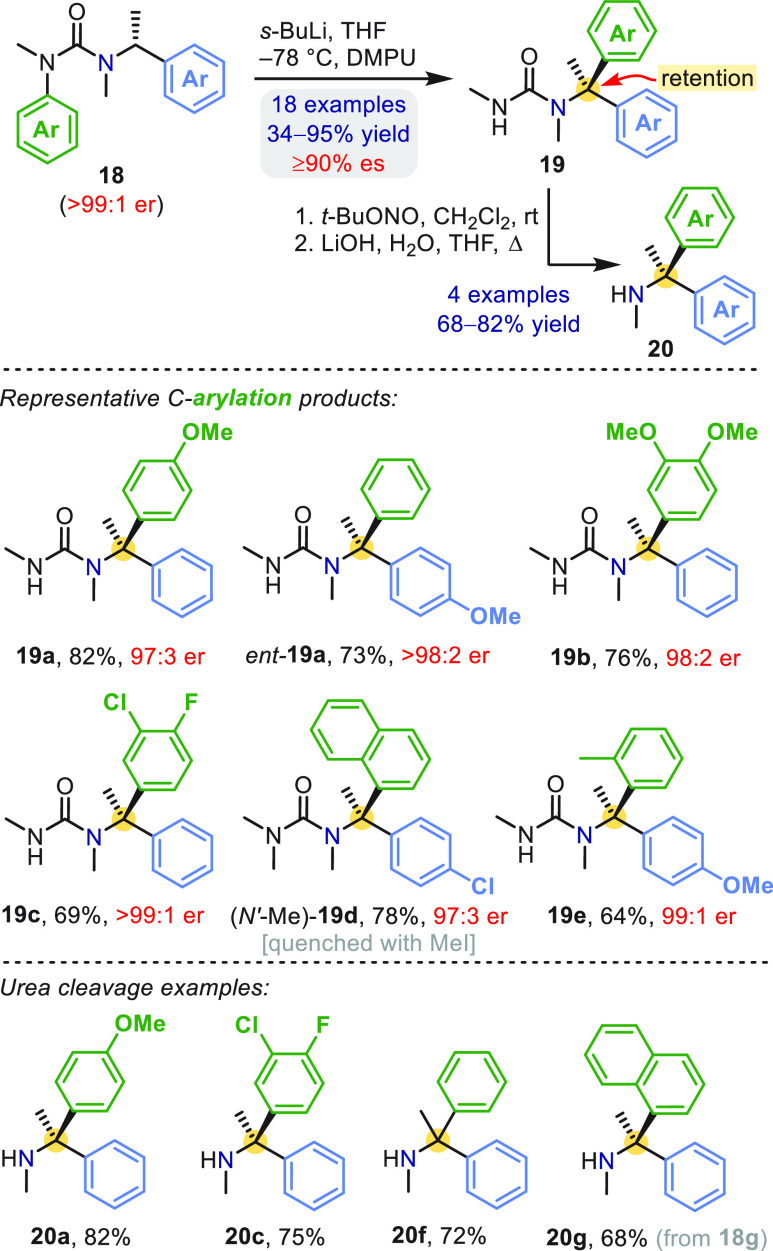
Enantiospecific Aryl Migration in α-Methylbenzylureas

Secondary benzylic ureas related to **15** underwent analogous rearrangement with high efficiency.^2^ The fact that complete aryl migration routinely occurred within 30 min at −78 °C led us to question whether such a process might be stereospecific with tertiary benzylic organolithiums. Indeed, with the addition of *N*,*N*′-dimethylpropyleneurea (DMPU) to accelerate carbanion arylation, a range of enantiopure α-methylbenzylureas **18** were rearranged to diarylalkylureas **19** with high enantiospecificity and net stereoretention (Scheme 4),^2^ including products bearing electron-rich (**19b**), electron-poor (**19c**), and sterically demanding arenes (**19d**, **19e**). The corresponding diarylamine derivatives **20** were accessible by hydrolysis of **19** after initial activation by *N*-nitrosylation (although we later discovered simpler conditions for this reaction; see below).

**Scheme 5:**
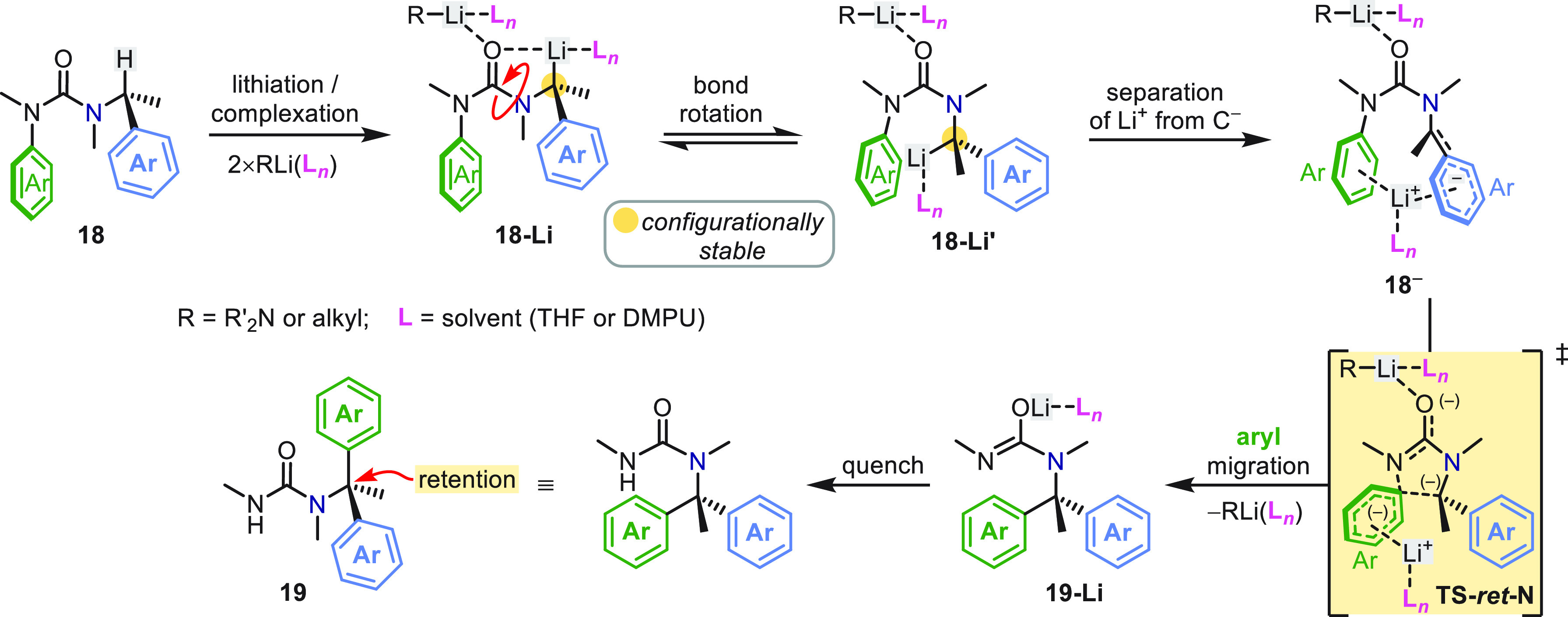
Mechanism of Stereoretentive Aryl Migration in α-Methylbenzylureas Supported by Computational Studies

A more complete mechanistic picture of the conversion of **18** into **19** emerged from density functional theory (DFT) computational studies (Scheme 5).^39^ First, carbonyl-directed benzylic lithiation provides **18-Li**, which undergoes rotation about the indicated N–CO bond to give reactive conformer **18-Li′**, which is primed for intramolecular S_N_Ar. Migration of the solvated lithium cation between the aryl rings in **18**^**–**^ leaves behind a delocalized benzylic carbanion, which retains transient axial chirality about the C^–^–N bond due to its perpendicular carbanion and urea planes. The reaction then passes through a low barrier, spirocyclic transition state (**TS-***ret***-N**) where the lithium cation stabilizes the building negative charge on the *N*′-aryl ring, resulting in 1,4-N → C aryl transfer with retention of configuration. The role of excess organolithium (RLi, ≥2 equiv) is, first, to promote decomplexation of the intramolecular O–Li interaction in **18-Li** through steric and electronic influence, which in turn promotes N–CO bond rotation, and, second, to stabilize the developing negative charge on the urea oxygen in **TS-***ret***-N**. In a complementary manner, DMPU (or THF) assists the rotation of **18-Li** to **18-Li′** and stabilizes **18**^**–**^ by coordination to the lithium cation. NMR and IR reaction monitoring failed to detect a dearomatized Meisenheimer intermediate between **18-Li** and **19-Li**, except in special cases where the migrating arene was a 1-naphthyl group.^2,39^

### 2.2 Other Lithiated *N*-Benzyl-, *N*-Allyl-, and *N*-Vinylureas: Expanding Scope

**Scheme 6:**
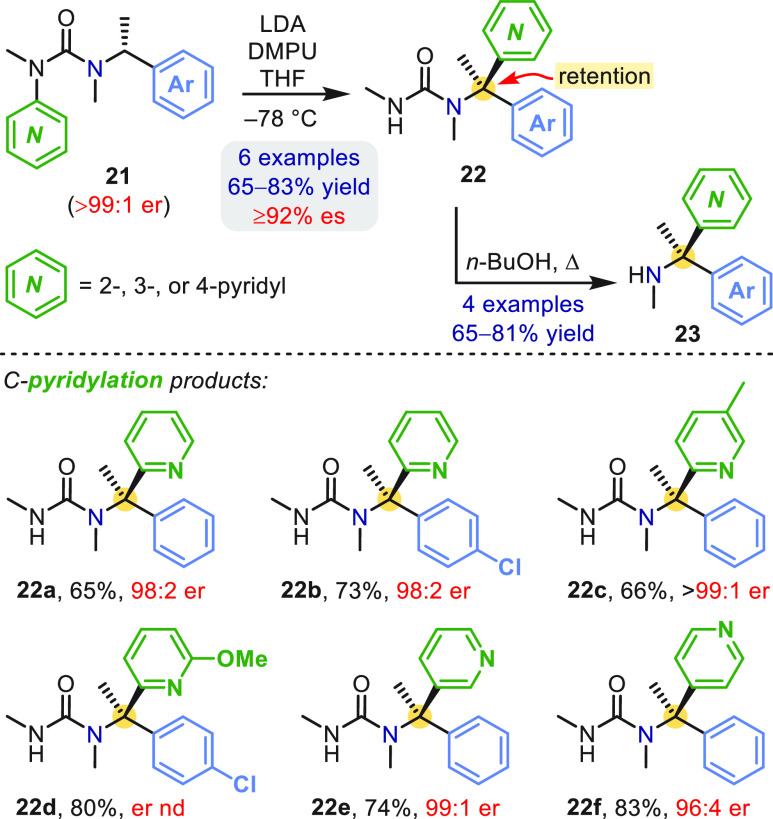
N → C Pyridyl Migration in α-Methylbenzylureas

A simple modification of our original conditions, using LDA as base instead of *s*-BuLi to avoid direct nucleophilic addition to the pyridine ring, enabled 2-, 3-, and 4-pyridyl groups to be transferred to the benzylic stereocenter in **21** with near complete enantiospecificity (Scheme 6).^40^ Solvolysis of the desired products **22** with *n*-BuOH revealed the corresponding amines **23** in representative cases: this method is equally applicable to other ureas and is now our method of choice for releasing the amine products.

**Scheme 7:**
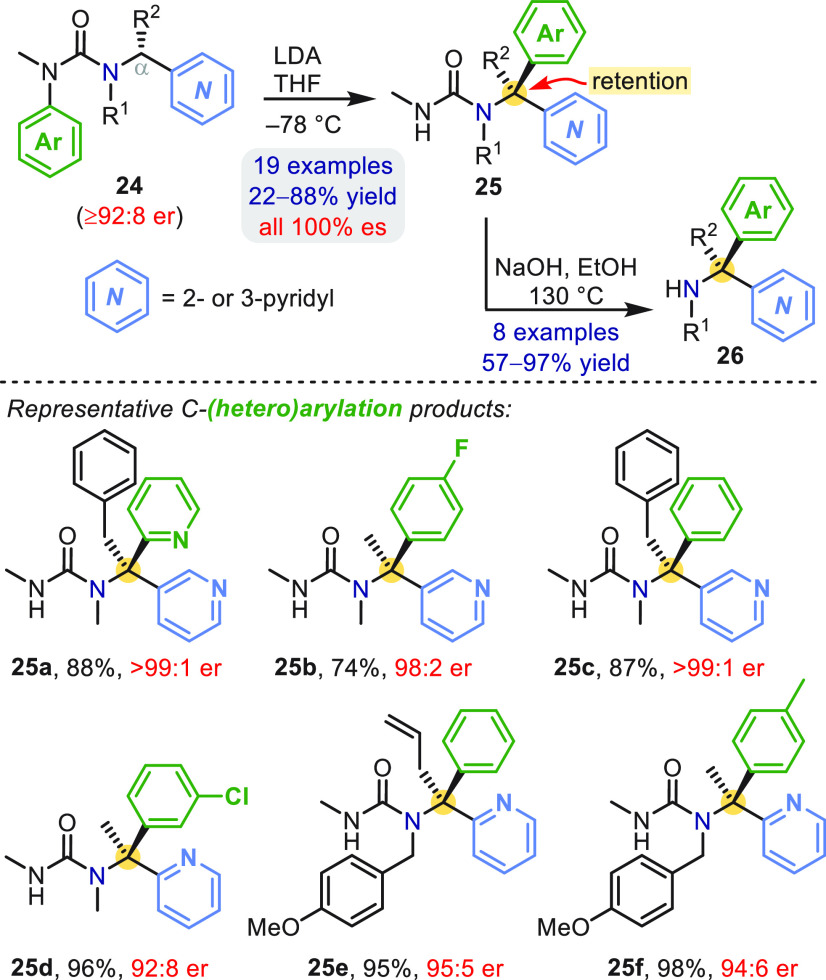
Aryl Migration in *N*-Pyridinylmethylureas

Enantioenriched α-pyridyl benzylamines **25** may also be made by stereoretentive aryl migration using pyridines as the anion stabilizing group (Scheme 7).^41^ The increased C–H acidity α to the pyridine ensured complete site selectivity in the initial deprotonation, even with allyl, benzyl, and *p*-methoxybenzyl (PMB) groups as R^1^ or R^2^. Even 2-pyridyl substrates **24** reacted enantiospecifically, despite the intermediacy of an aza-enolate-like species, which must preserve homochirality on the time scale of the aryl transfer.^41^ Amine derivatives **26** were isolated in good yields after urea cleavage, this time with hydroxide.

**Scheme 8:**
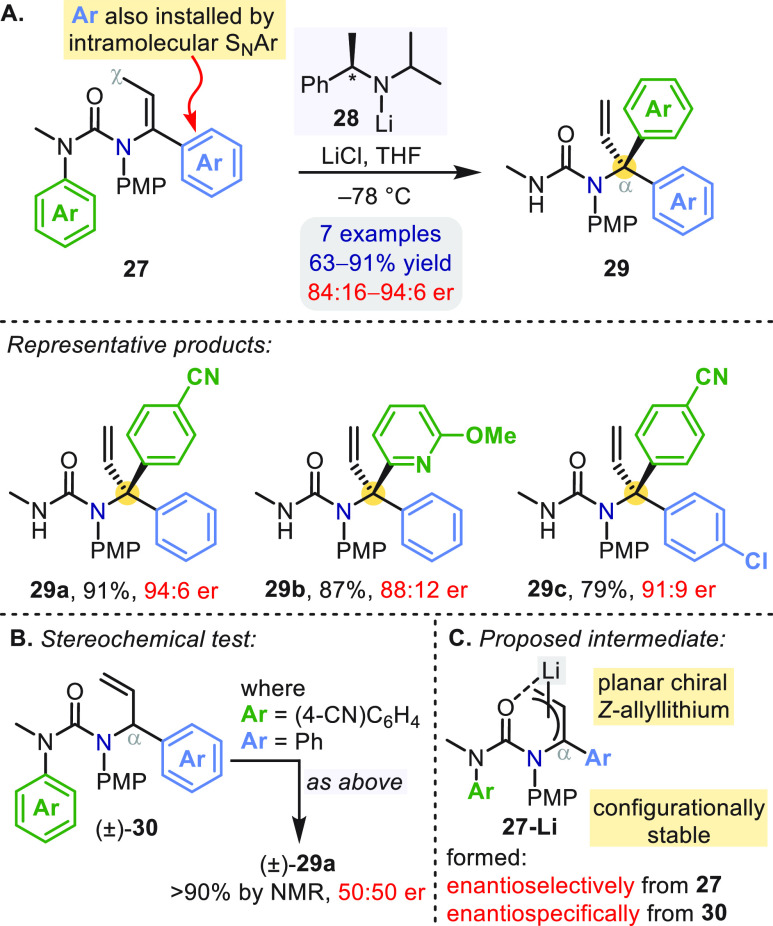
Enantioselective Aryl Migration in *N*-Vinylureas

With allyllithiums as the carbon nucleophiles in the intramolecular S_N_Ar reactions, two different aryl groups could be transferred in succession to the α-carbon of *N*-allyl ureas.^42^ LDA-promoted N′ → C_α_ aryl migration was followed by a palladium-catalyzed *N*-arylation of the urea to give the intermediate ureas **27** (Scheme 8a). A second C_α_-arylation (involvingthe green aryl ring) was effected by the chiral lithium amide **28** to give enantioenriched α,α-diaryl allylic amine derivatives **29**. To probe the enantiodetermining step in the conversion of **27** to **29**, an isomeric substrate (±)-**30** (Scheme 8b) was used as an alternative starting point for the reaction via the same allyllithium intermediate. The racemic product (±)-**29a** ruled out the interconversion of diastereomeric allyllithium·**28** complexes as the stereodetermining step. δ-Deprotonation of **27** by **28** presumably gives directly an enantioenriched, configurationally stable planar chiral allyllithium **27-Li** (Scheme 8c), which undergoes stereospecific α-arylation.

**Scheme 9:**
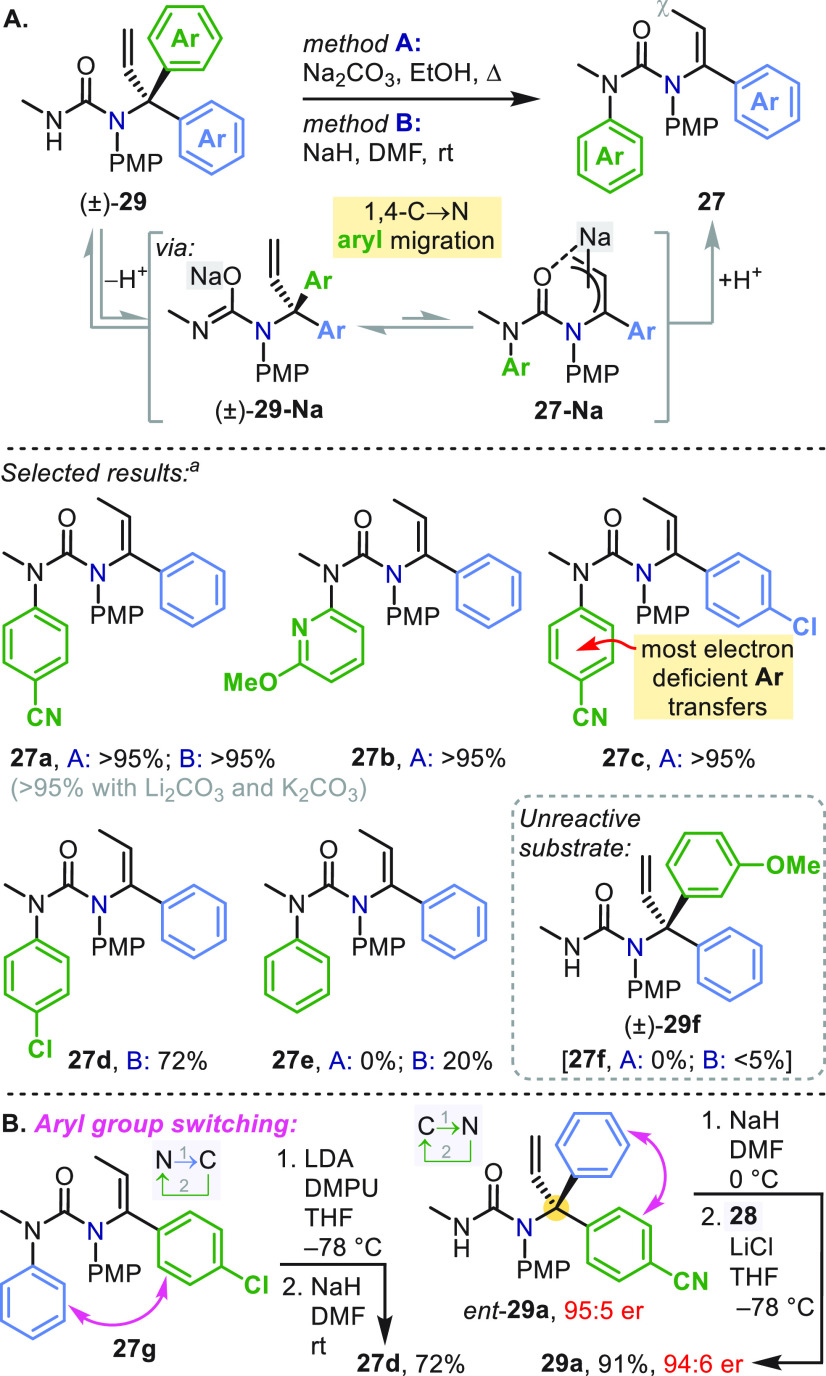
Reversible Aryl Migrations Yields for methods A and B are NMR yields.

An unexpected discovery was made during hydrolysis attempts using Na_2_CO_3_/EtOH or NaH/DMF (Scheme 9a): with an electron-deficient arene in **29**, “reverse” 1,4-aryl migration (C → N) occurred to return vinyl ureas **27**.^43^**29-Na** presumably exists in a rapid but unfavorable equilibrium with **27-Na** by C → N aryl migration, and irreversible γ-protonation of **27-Na** by the solvent or remaining **29** drives the reverse rearrangement to completion.

This newly uncovered reactivity made possible some remarkable molecular reorganizations using a cycle of N → C → N or C → N → C aryl shuttling (Scheme 9b), exchanging the arene constitution in vinyl ureas **27g**/**27d** or inverting the configuration of urea **29a**.^43^

**Scheme 10:**
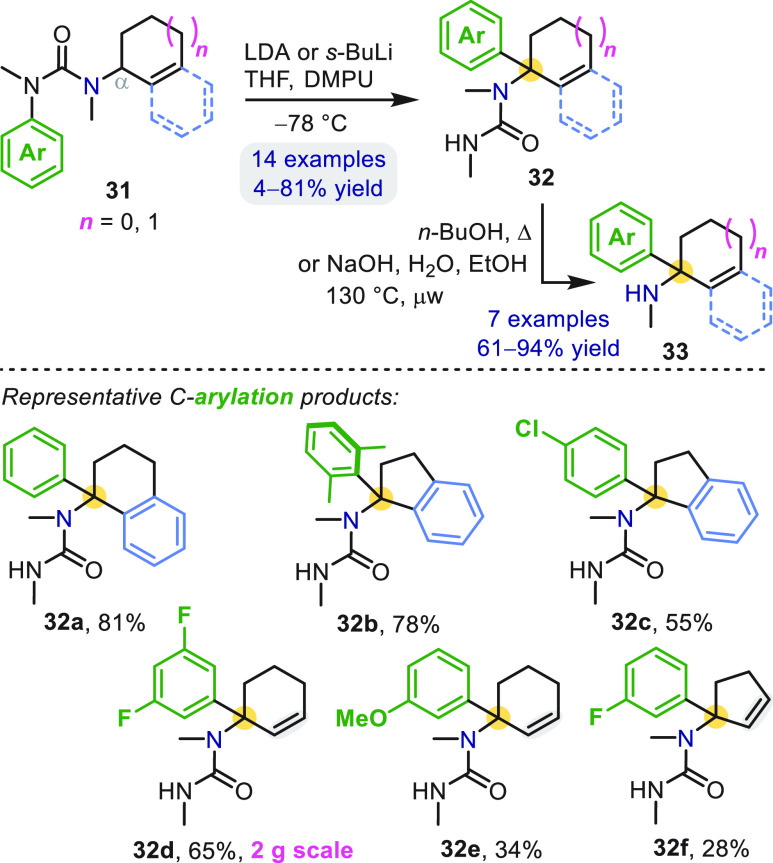
Aryl Migration in Ureas Derived from Exocyclic Amines

**Scheme 11:**
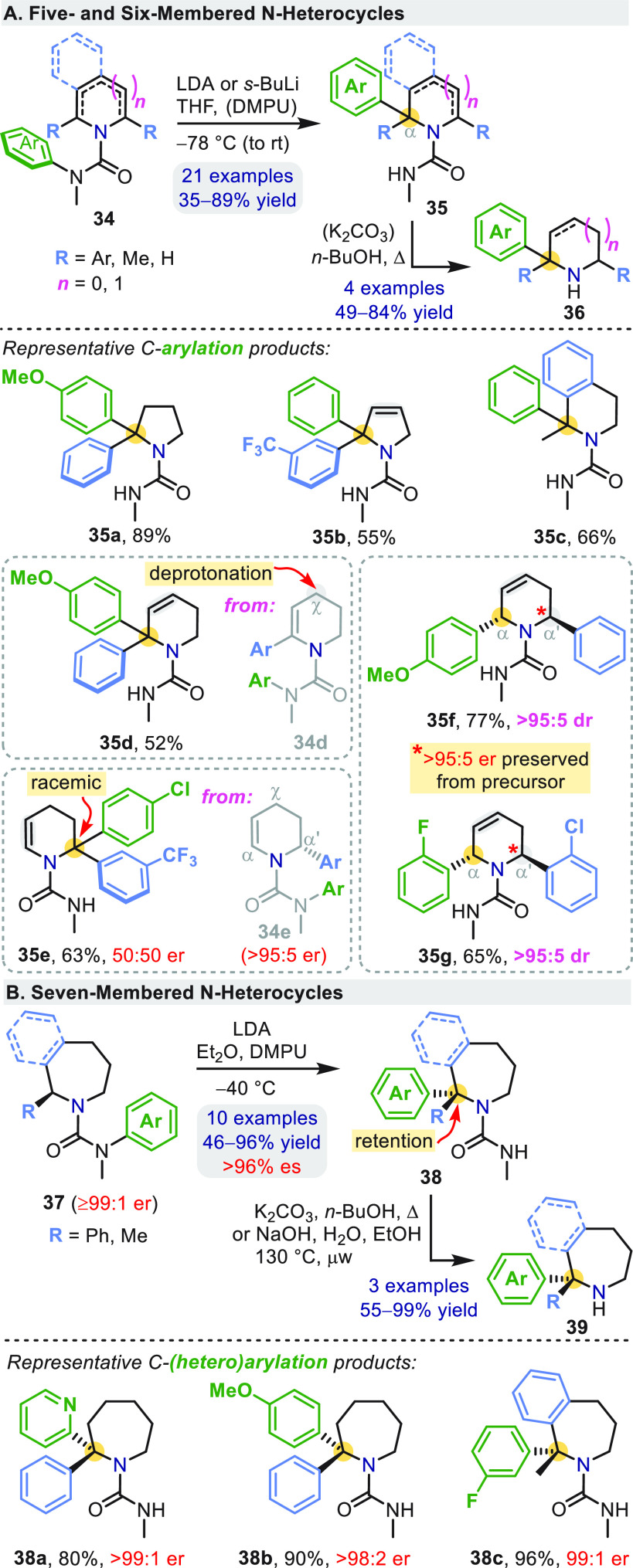
Aryl Migration in Ureas Derived from Endocyclic Amines

In substrates **31**, the nucleophilic allylic or benzylic α-carbon is part of a five- or six-membered carbocycle (Scheme 10).^44,45^ These give α-aryl exocyclic amine derivatives **32** containing electron-rich (**32b**) and electron-poor (**32f**) arenes, as well as structures (**32d** and **32e**) closely related to the anesthetic ketamine (**4**, Figure 1). Similarly, heterocyclic *N*′-aryl ureas **34** underwent efficient α-arylation when treated with base (Scheme 11a).^45,46^ High regioselectivity in the formation of **35e**–**35g** arises from the kinetic preference of deprotonation by the bulky base (α-allylic > α′-benzylic > γ-allylic), rather than differing reactivities of equilibrating organolithiums. This is supported by the preservation of enantiopurity in **35f** and **35g**, confirming that no proton exchange occurred at the existing benzylic α′-stereocenter before migration of the second (green) arene.

Rearrangements involving the five- and six-membered cyclic systems **31** and **34** were not stereospecific at the lithiation site: enantioenriched samples of **31c** or **34e** gave racemic **32c** and **35e** (Schemes 10 and 11a).^45,46^ Slower rearrangement when the carbon nucleophile is within such a ring as a consequence of the more strained bicyclic transition state appears to allow racemization of the organolithium to outcompete C–C bond formation. By contrast, enantiospecificity of the arylation is restored on moving to seven-membered ring systems **37** (Scheme 11b), which give α,α-diaryl azepanes **38** in enantiopure form.^47^

**Scheme 12:**
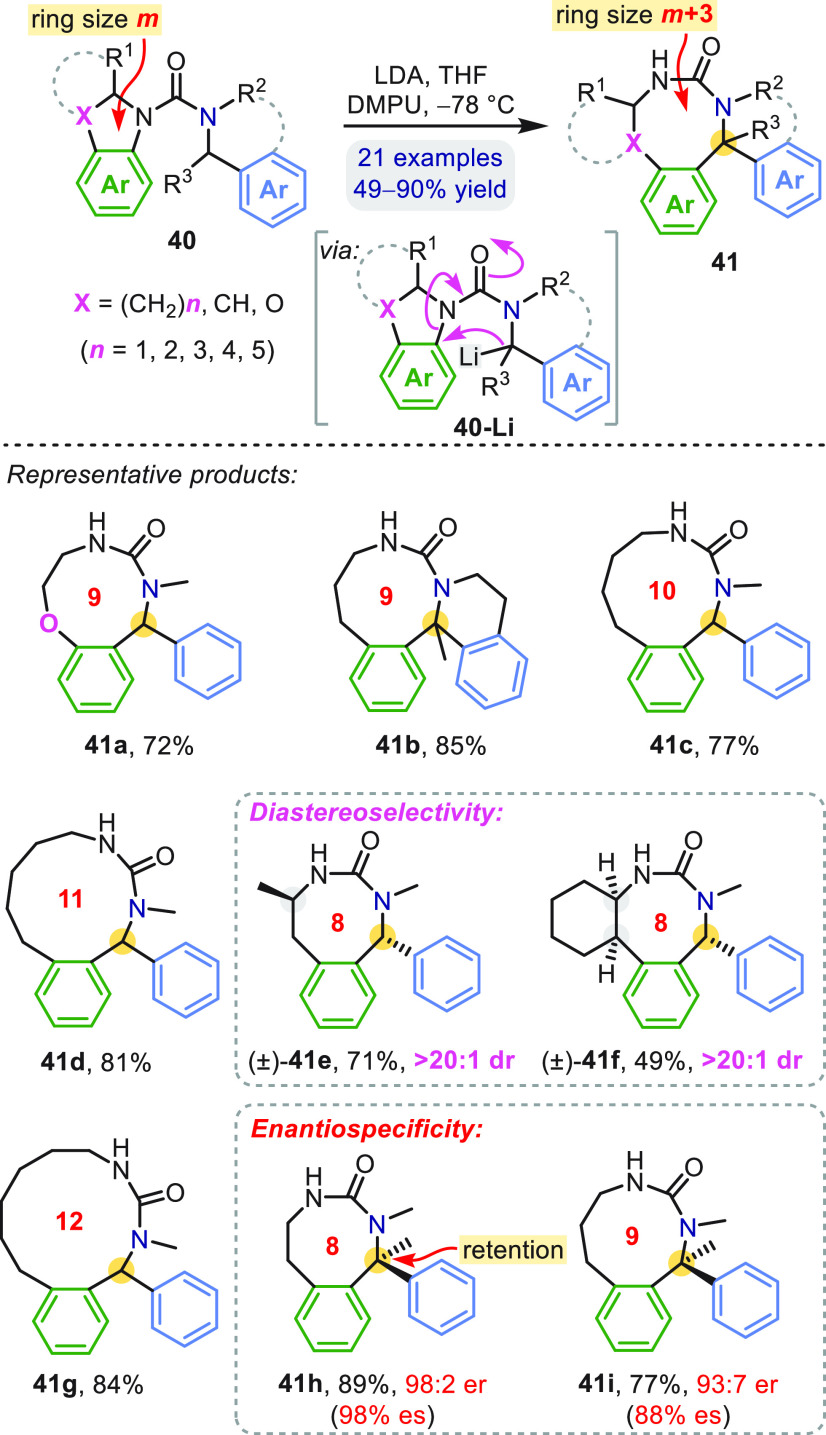
Ring-Expanding Aryl Migration

With the migrating *N*′-aryl electrophile embedded in a heterocyclic system, migration leads to a three-atom ring expansion, giving medium rings **41** (Scheme 12).^48^ Urea derivatives **40** of a variety of common *N*-heterocycles (e.g., indoline, tetrahydroquinoline, benzomorpholine, benzoazepine) provided starting materials for a practical synthesis of 8- to 12-membered heterocycles. The established attributes of the T-S rearrangements described previously are exhibited by this ring expanding variant: unactivated arenes function as migrating groups, the carbon nucleophile may be cyclic (**41b**) or acyclic, and the reaction is both highly diastereoselective (**41e**, **41f**) and enantiospecific (**41h**, **41i**).

In an interesting synthetic application (not shown), eight- to ten-membered heterocycles **41** underwent an acid-promoted S_N_1-like rearrangement at the benzylic carbon, in which the proximal urea NMe nitrogen was displaced by the distal urea NH.^49^ Overall, a three-atom ring expansion (Scheme 12) and ensuing two-atom ring contraction constitute the formal insertion of a benzylic carbon into the C_aryl_–N bond of a nitrogen heterocycle.

### 2.3 Enolates and Metalated Nitriles: Synthesis of Hydantoins and Quaternary Amino Acids

**Scheme 13:**
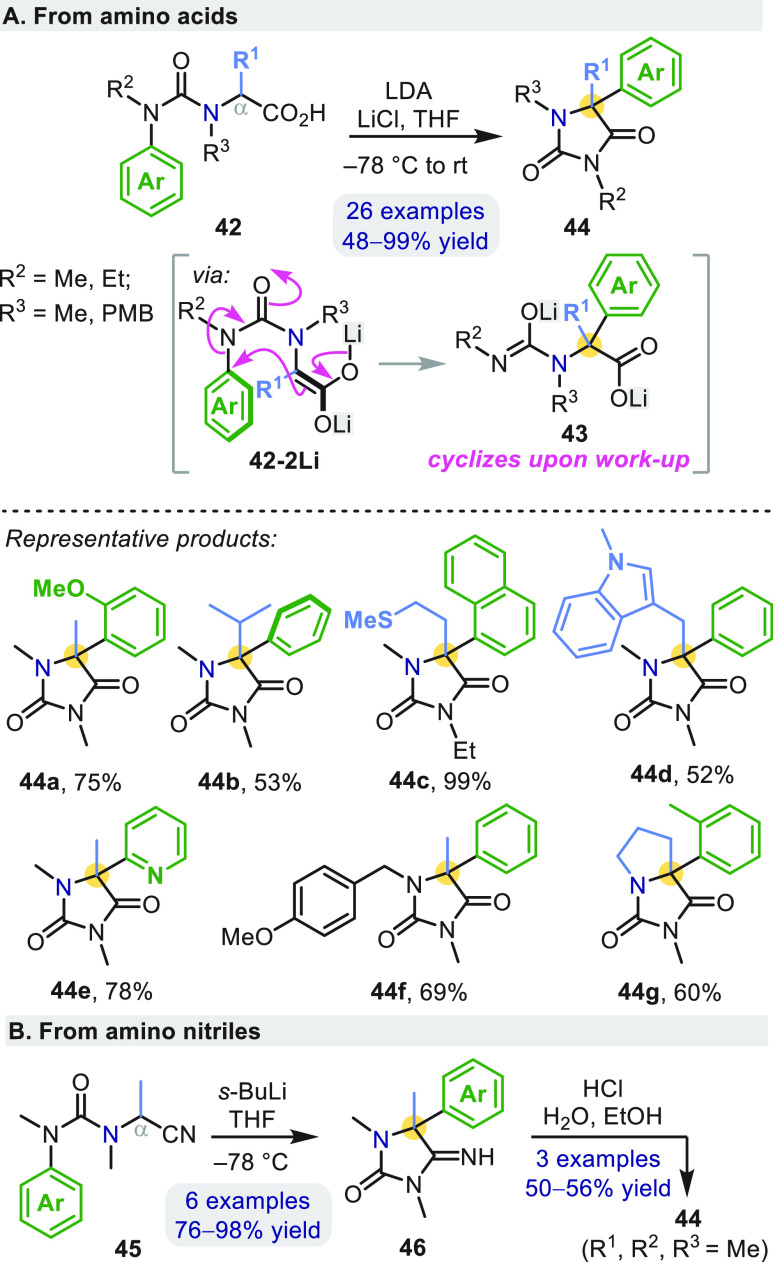
Aryl Migration in Ureas Derived from Amino Acids and Nitriles

The success of intramolecular S_N_Ar processes with carbanions as nucleophiles encouraged us to investigate rearrangements of other, less basic carbon nucleophiles such as enolates. For example, treating amino acid-derived ureas **42** with LDA and LiCl likewise led to C_α_-arylation under mild conditions (Scheme 13a).^50^*In situ* IR reaction monitoring showed lithium carboxylate **42-Li**, dianionic enolate **42-2Li**, and dianion **43** as the sole reaction intermediates; spontaneous cyclization of **43** to **44** occurs upon quenching with MeOH. Cleavage of the PMB group in **44f** facilitated alkaline hydrolysis to the corresponding α-quaternary amino acid.^50^

**Scheme 14:**
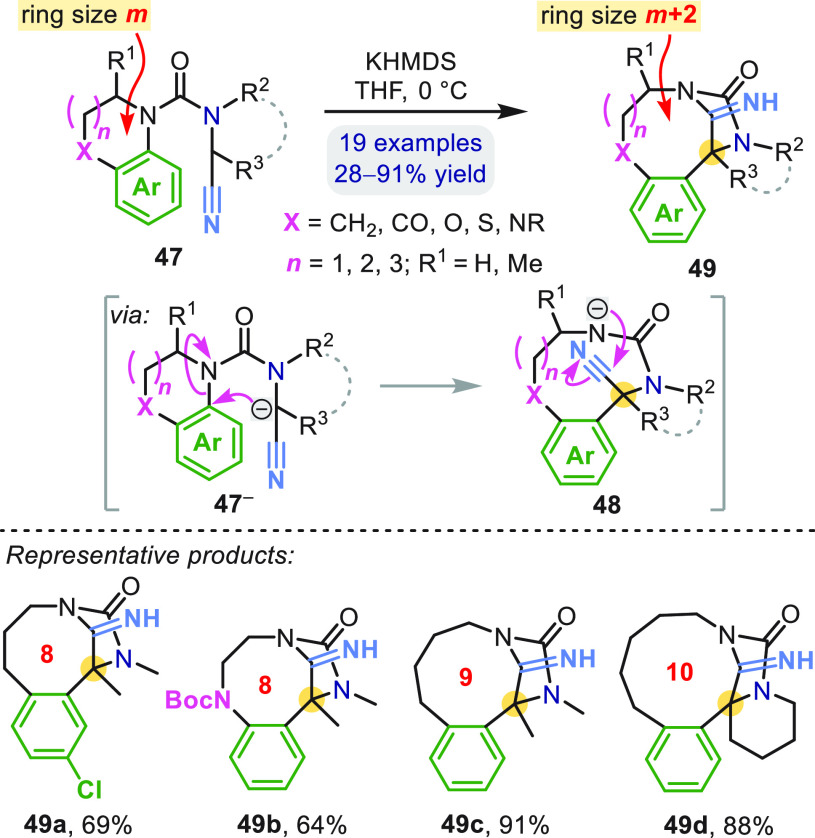
Ring-Expanding Aryl Migration to Give Hydantoin-Bridged Medium Rings

Hydantoins **44** also form from amino nitrile-derived ureas **45** (Scheme 13b).^50^ The iminohydantoin products of the rearrangement **46** hydrolyzed to **44** with acid. Related nitrile-stabilized carbanions **47**^**–**^ allowed ring expansion of heterocycles **47** to iminohydantoin-bridged eight- to ten-membered N-heterocycles **49** (Scheme 14).^51^ When X was (or was part of) a pronucleophile (e.g., CO or NBoc as in **49b**), a second transannular *exo*-cyclization onto the C=N bridge formed even more complex caged structures.^51^

**Scheme 15:**
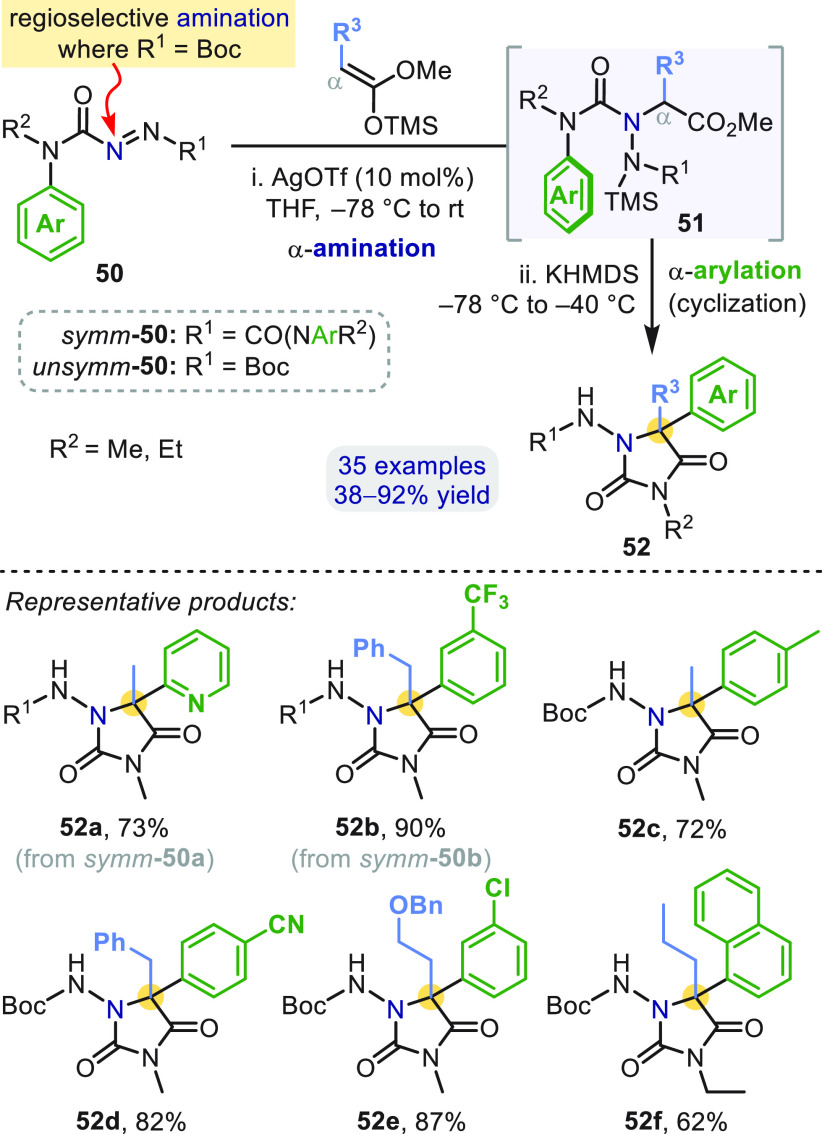
Hydantoins by Tandem α-Amination/α-Arylation of Silyl Ketene Acetals

The synthesis of α-aryl hydantoins by enolate arylation was streamlined into a one-pot sequential α-amination and α-arylation of silyl ketene acetals (Scheme 15).^52^ AgOTf-catalyzed α-amination of the masked ketene by **50** gives α-amino ester **51**, from which potassium hexamethyldisilazide (KHMDS) triggers *N*-desilylation to an ester enolate to which the aryl group migrates, releasing the urea, which cyclizes onto the ester to give hydantoins **52**. The overall transformation in Scheme 15 may be considered a formal (3 + 2)-cycloaddition, where **50** serves as a latent “N^–^–C(=O)–N^+^” 1,3-dipole.

**Scheme 16:**
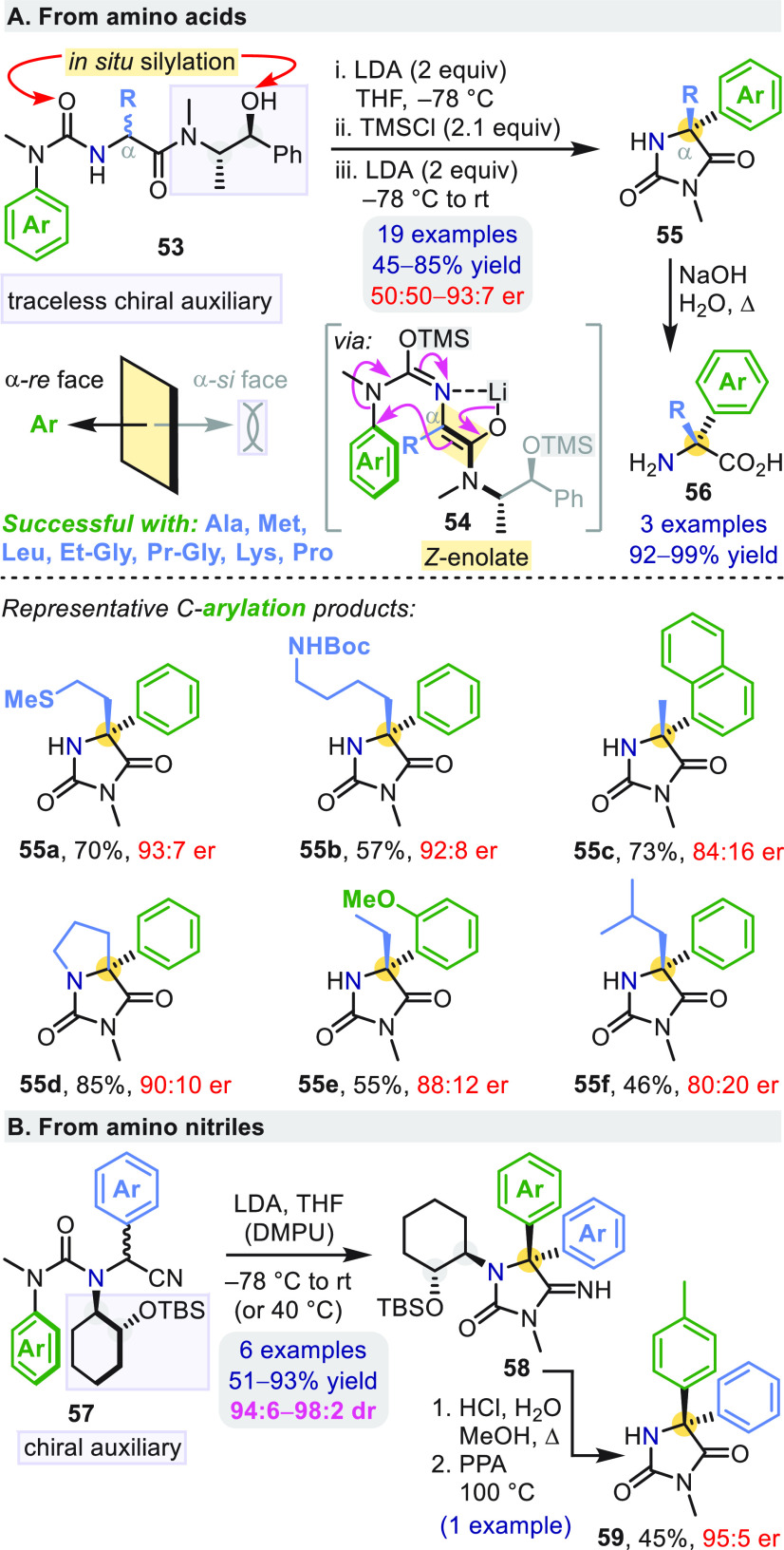
Chiral Auxiliary-Directed Arylation of Amino Acids and Nitriles

Asymmetric enolate arylation was initially achieved using chiral auxiliaries, among which pseudoephedrine proved most effective (Scheme 16a).^53^ Trisubstituted urea **53** was silylated *in situ* (steps i and ii) prior to generation of enolate **54** (step iii). Arylation was followed by spontaneous cyclization of the anionic urea, expelling the recyclable pseudoephedrine auxiliary and providing enantioenriched quaternary hydantoins **55**.

Enolates of phenylglycine-derived substrates were too unreactive under these conditions, so a chiral cyclohexyl auxiliary was instead appended to the nitrogen of an amino nitrile-derived substrate, **57** (Scheme 16b).^54^ This modified approach enabled the enantioselective synthesis of the previously elusive chiral (imino)phenytoin analogues **58** and **59**.

**Scheme 17:**
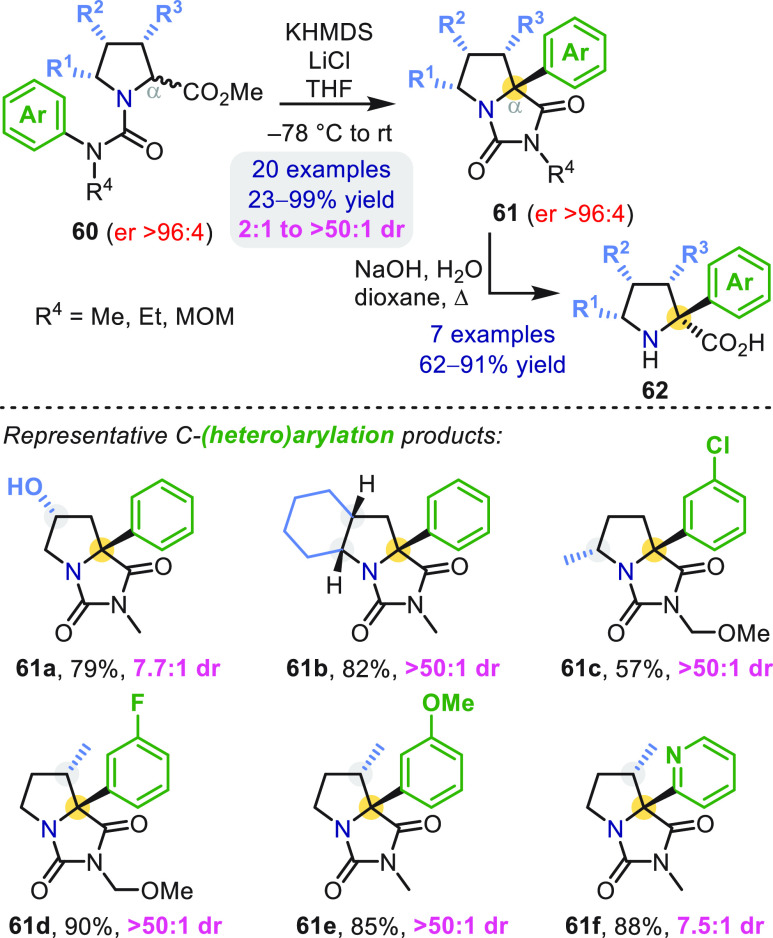
Diastereoselective Aryl Migration in Proline-Derived Ureas

Stereocenters within a urea substrate also direct the facial selectivity of enolate arylation (Scheme 17). Enantioenriched ureas **60** with defined backbone chirality were treated with KHMDS to generate a set of α-aryl proline derivatives **61** and **62** as single diastereomers in most cases.^55^ Unlike related heterocycles **35f** and **35g** (Scheme 11), the α-carbon in **61** is fully substituted, so epimerization by further deprotonation is not possible and the diastereoenrichment in **61** must reflect the kinetic selectivity of C-arylation. It is therefore noteworthy that high substrate-controlled diastereoselectivity was observed regardless of whether the directing stereocenter was at the 3-, 4-, or 5-position of the heterocycle backbone (**61a**–**61d**).

**Scheme 18:**
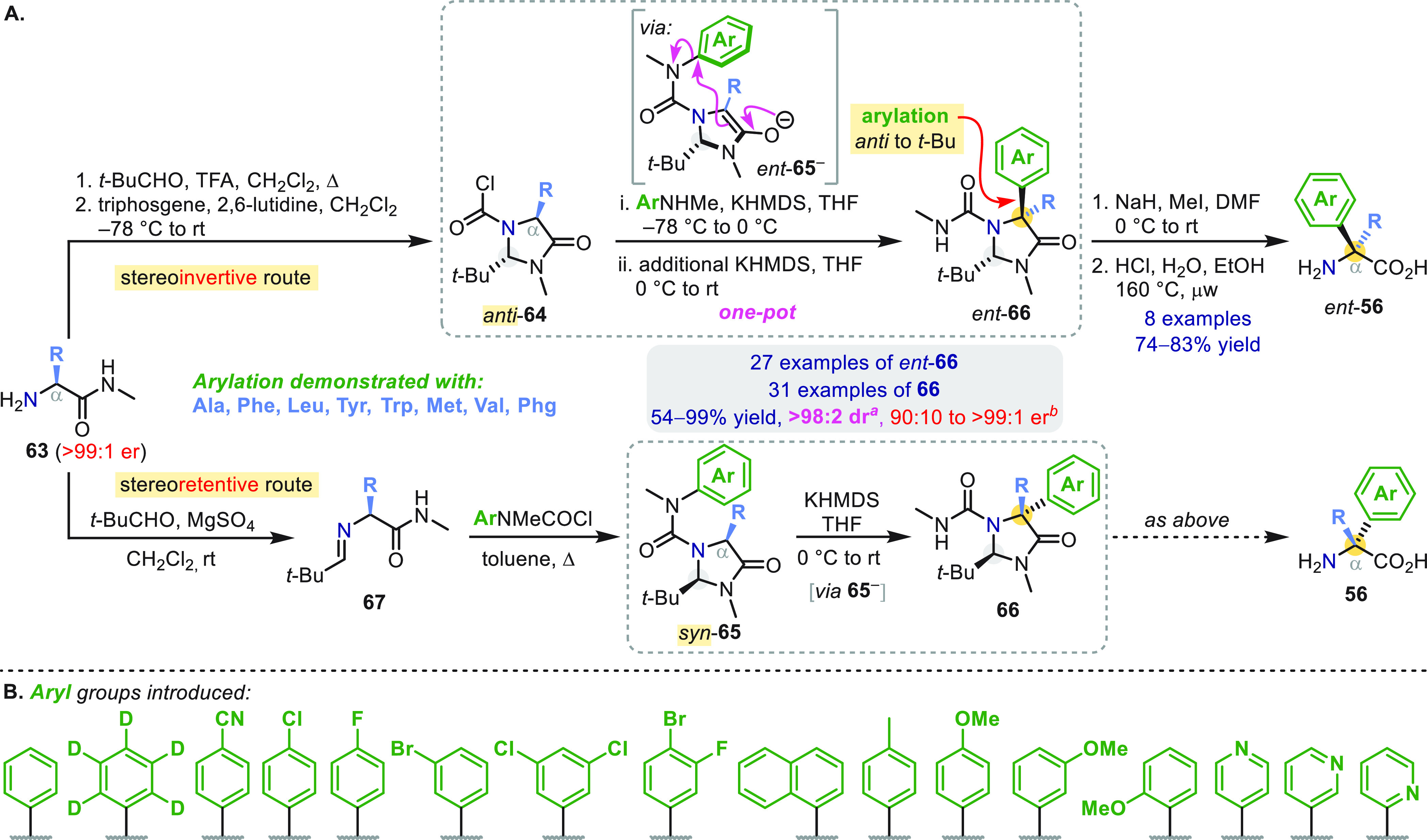
Asymmetric Arylation of Amino Acids by Self Regeneration of Stereocenters One exception was a valine-derived substrate, which gave 91:9 dr using Et_2_NLi (stereoretentive route). An exception was phenylglycine-derived substrates via the stereoretentive route, which gave 81:19–87:13 er values due to partial racemization during conversion of **67** to *syn*-**65**.

The diastereocontrol displayed by cyclic enolates found its most useful application in an asymmetric arylation of acyclic amino acids based on Seebach’s “self-regeneration of stereocenters” concept (Scheme 18a).^1,56^ Starting from amino acid derivatives **63**, either the *anti* or *syn* epimer of the required imidazolidinone could be formed, ultimately enabling both α-arylated enantiomers of **56** to be prepared from l-amino acids. α-Deprotonation of urea *anti*-**65**, formed *in situ*, or *syn*-**65** transiently erases the α-stereocenter to give a pair of enantiomeric enolates *ent*-**65**^**–**^ (shown) and **65**^**–**^ that undergo diastereoselective arylation *anti* to the *t*-Bu directing group. The configuration of the newly arylated α-stereocenter is thus inverted in *ent*-**66** and retained in **66**.

The broad utility of this C_α_-arylation (Scheme 18a) was demonstrated by 58 examples of the synthesis of **66** and *ent*-**66** from a pool of eight different amino acid precursors (listed) and 16 different migrating (hetero)arenes exhibiting the full range of electronic characters (Scheme 18b).^1^ A Hammett kinetic analysis of the conversion of *syn*-**65** to **66**, followed by *in situ* IR spectroscopy, revealed that enolate formation was rate-determining for electron-poor arenes, while enolate arylation was rate-determining for electron-rich arenes. In the latter domain, a ρ value of +4.5 was obtained, consistent with a partially concerted S_N_Ar mechanism.^31−34^ Enantiopure α-aryl quaternary amino acids *ent*-**56** were formed by a straightforward sequence of *N*-methylation (required to avoid hydantoin formation) and acidic hydrolysis (Scheme 18a).

**Scheme 19:**
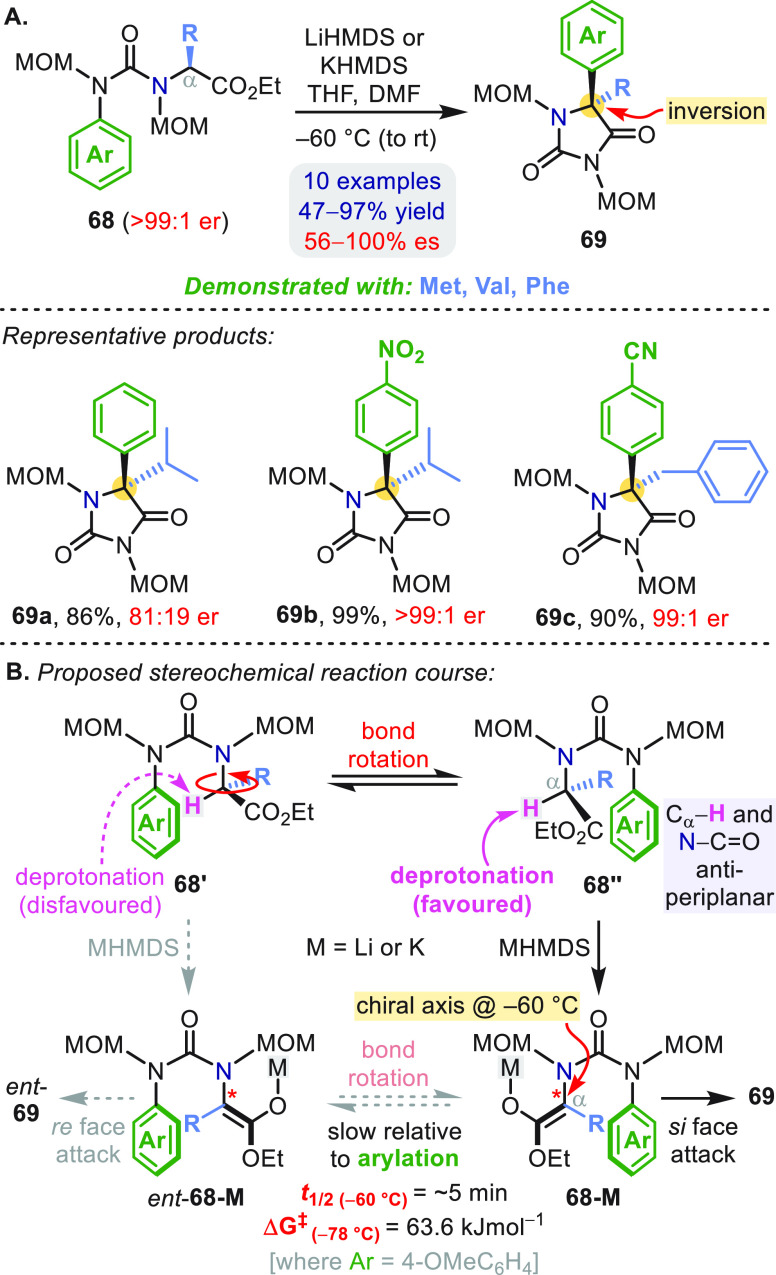
Arylation of Amino Acids via Memory of Chirality

A different “chiral memory” approach to the asymmetric α-arylation of a group of amino acids with bulky side chains was reported by Kawabata using urea-substituted axially chiral enolates (Scheme 19a).^57^ Deprotonation of amino esters **68** at −60 °C resulted in a stereoinvertive α-arylation to give hydantoins **69**. Mechanistically, selective deprotonation of **68″** (over diastereomeric conformer **68′**) was proposed as a result of its antiperiplanar C_α_–H and urea N–CO bonds (Scheme 19b), forming an enantioenriched *Z*-enolate **68-M** that must attack the aryl ring from its α-*Si* face due to restricted rotation about the C_α_–N bond. Complete enantiospecificity was observed for electron-poor arenes (**69b**, **69c**) but slower arylation rates with less activated rings (**69a**) compromised the enantiopurity (Scheme 19a). Nonetheless, the fact that enolate **68-M** was arylated faster than racemization in certain cases is remarkable and reinforces the powerful conformationally activating effect of the urea tether.

## 3 N → C Aryl Migration in Carbamates: Synthesis of Tertiary Alcohols

**Scheme 20:**
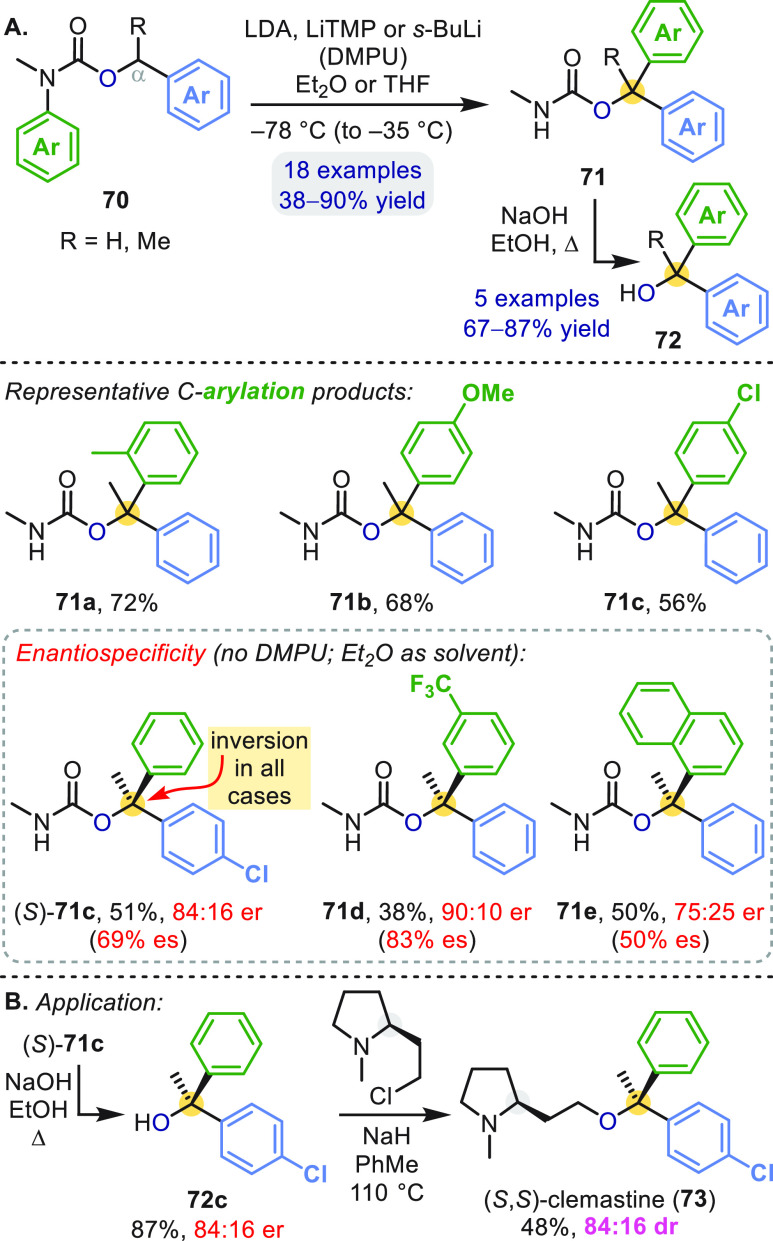
Aryl Migration in *O*-Benzylcarbamates

Like their urea congeners, *N*-aryl-*N*-alkyl carbamates exhibit a strong preference for a conformation in which the *N*-aryl and carbonyl groups lie *trans*. As a consequence, α-lithiation of *N*-aryl carbamates **70** likewise triggers a N → C transfer of a variety of aryl groups to give rearranged products **71** (Scheme 20a).^58^ The corresponding α,α-diaryl alcohols **72** may then be returned by hydrolysis with NaOH.

The rearrangement of enantioenriched carbamates **70** (R = Me) likewise gave enantioenriched arylation products (*S*)-**71c**–**71e** (Scheme 20a);^58−60^ but only moderate enantiospecificity (50–83% es) arose, due to racemization of the organolithium on the time scale of the arylation. Nonetheless, (*S*)-**71c** (84:16 er) provided a key intermediate in the first enantioselective synthesis of the antihistamine clemastine (Scheme 20b).^59^

**Scheme 21:**
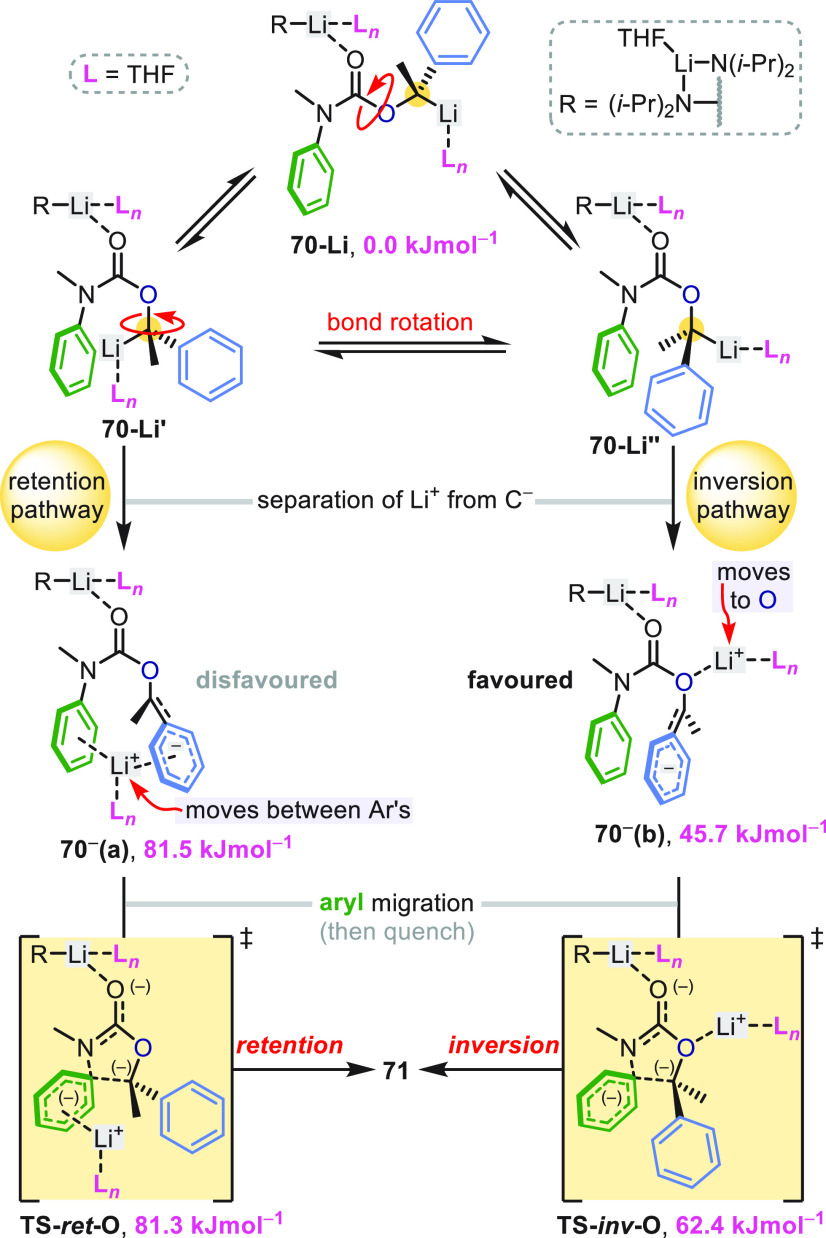
Stereoinvertive Aryl Migration in Lithiated Carbamates

A distinctive feature of the lithiated carbamates was the stereochemical course of their C-arylation: (*S*)-**71c**–**71e** were formed with *inversion* of configuration (Scheme 20a),^58−60^ in contrast to the stereochemical *retention* of related ureas (Schemes 4–7). DFT calculations of the T-S rearrangement of carbamate **70** (R = Me, both Ar = Ph) illuminated the origin of this effect. Scheme 21 shows two possible trajectories identified from **70-Li**: an energetically favorable “inversion” pathway, and a higher energy “retention” pathway^58,60^ analogous to that taken by ureas (Scheme 5).^39^ These pathways (Scheme 21) differ primarily in the direction that the lithium cation takes during charge separation from the carbanion and importantly they lead to opposite enantiomers because they start from different conformers of the benzyllithium (**70-Li**′ or **70-Li**″). The lowest energy pathway proceeds from **70-Li**″ to **70**^**–**^**(b)** where the lithium cation migrates to the α oxygen’s available lone pair. The opposite face of the carbanion is now available to attack the arene through the low barrier **TS-***inv***-O** (Δ*G*^⧧^ = 16.7 kJ mol^–1^), giving **71** with net configurational inversion. The (disfavored) retentive pathway for the carbamates is higher in energy than the analogous pathway calculated for ureas by ∼40 kJ mol^–1^,^39^ showing that carbamates give stereochemical inversion both by disfavoring the retention pathway and by opening up an inversion pathway unavailable to ureas, which lack a electron pair orthogonal to the C=O π bond.

**Scheme 22:**
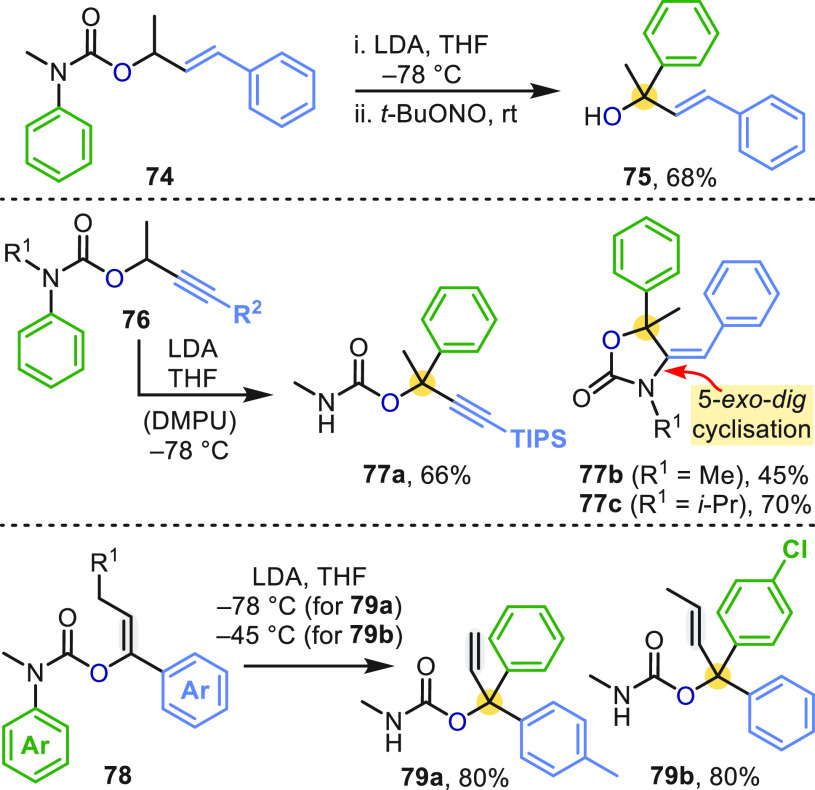
Aryl Migration in *O*-Cinnamyl-, *O*-Propargyl-, and *O*-Vinylcarbamates

*O*-Cinnamyl-, *O*-propargyl- and *O*-vinylcarbamates (**74**, **76**, and **78**) all underwent T-S rearrangements when treated with lithium diisopropylamide (LDA) (Scheme 22).^60^ In the case of propargyl substrates **76** bearing a terminal phenyl group (R^2^ = Ph), the aryl migration was followed by spontaneous 5-*exo*-*dig* cyclization of the urea nucleofuge to give oxazolidinones **77b** and **77c**.

## 4 N → C Aryl Migration in *S*-Thiocarbamates: Synthesis of Tertiary Thiols

**Scheme 23:**
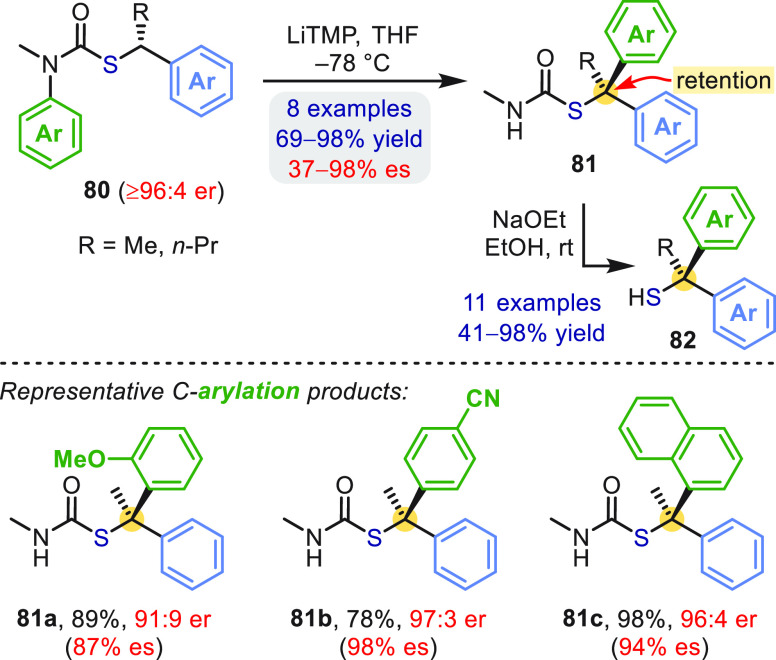
Enantiospecific Aryl Migration in α-Alkylbenzylthiocarbamates

Chiral, nonracemic tertiary thiols are challenging synthetic targets, but conformationally activated T-S rearrangements also provide an enantioselective entry to this compound class. As summarized in Scheme 23, the addition of lithium tetramethylpiperidide (LiTMP) to enantioenriched *S*-benzylic thiocarbamates **80** resulted in N → C migration of electronically diverse arenes to give tertiary thiol derivatives **81** with generally high stereospecificity (≥87% es) and ≥91:9 er.^61^ Only when the nucleophilicity of the intermediate benzyllithium was attenuated (blue Ar = 3-CF_3_C_6_H_4_) was the enantiopurity of **81** compromised by significant racemization. The corresponding enantioenriched α,α-diaryl thiols **82** were obtained simply by stirring **81** with NaOH in EtOH at room temperature for 15 min.

**Scheme 24:**
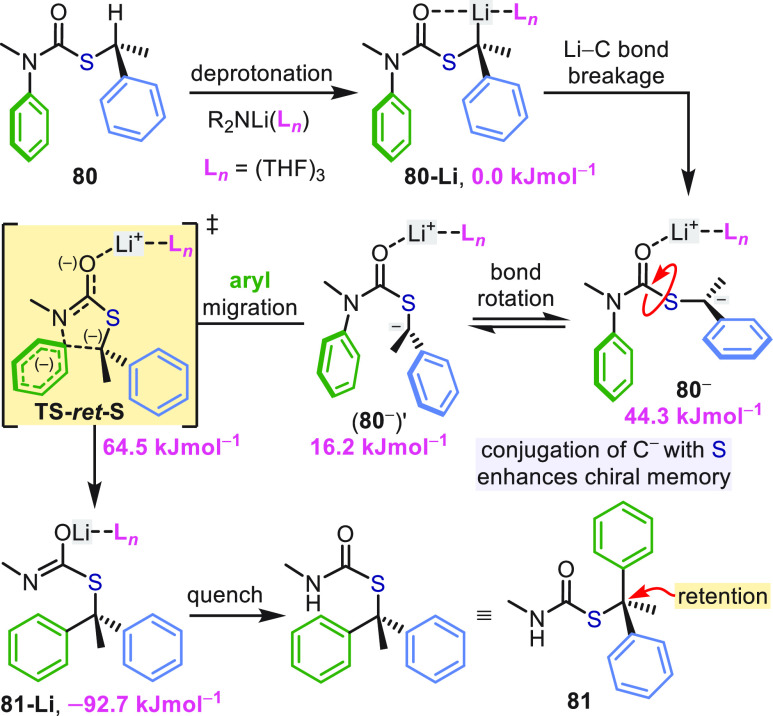
Stereoretentive Aryl Migration in Lithiated Thiocarbamates

As with benzylic ureas, the configuration of the stereocenter in *S*-thiocarbamates **80** was retained upon arylation (Scheme 23).^61^ Nonetheless, a unique mechanistic trajectory for the T-S rearrangement of **80** (R = Me, both Ar = Ph) was identified by DFT calculations (Scheme 24).^62^ Previous experimental and theoretical investigations of the T-S rearrangements of both ureas and carbamates showed that population of the reactive organolithium conformation for S_N_Ar requires coordination of exogenous lithium base to drive X–CO bond rotation (Schemes 5 and 21).^39,60^ Evidently, this is not the case for *S*-thiocarbamates **80** (Scheme 24); instead, the stabilizing effect of sulfur on the α carbanion allows C–Li bond cleavage to occur directly from the intramolecularly complexed **80-Li**, accompanied by a 1,4-shift of the lithium cation to the carbonyl oxygen. The consequence is that S–CO bond rotation in **80**^**–**^ on route to the reactive conformer **(80**^**–**^**)′**, which lacks a C–Li bond, is now energetically favorable. Meanwhile, partial π-character to the C^–^–S bond in the planar carbanion **80**^**–**^ provides a form of chiral memory, preventing C^–^–S bond rotation and stereochemical scrambling. As such, the same face of the carbanion **(80**^**–**^**)′** is presented for attack on the arene as originally occupied by lithium, resulting in retention of configuration via **TS-***ret***-S** (Δ*G*^⧧^ = 48.3 kJ mol^–1^).^62^

**Scheme 25:**
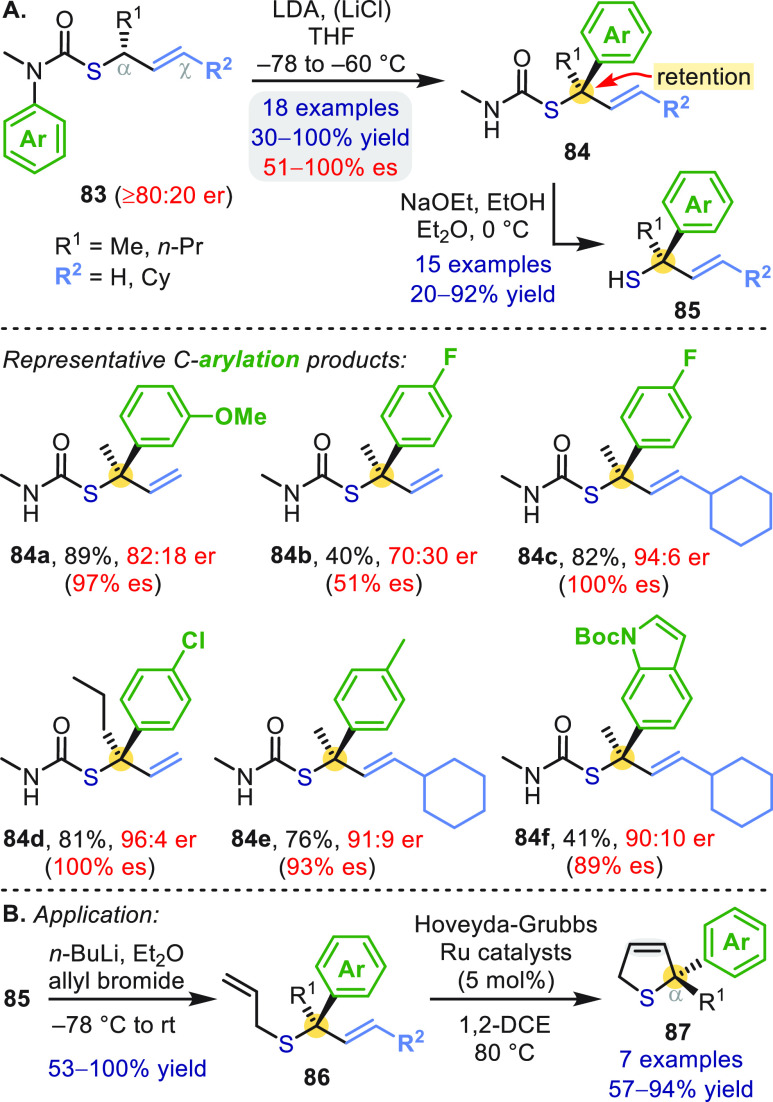
Enantiospecific Aryl Migration in Allylthiocarbamates

Tertiary allylic thiols were synthesized by way of enantioenriched thiocarbamates **83** (Scheme 25a), formed by enantioselective (R^2^ = H)^63^ and enantiospecific (R^2^ = Cy)^64^ [3,3]-sigmatropic rearrangements of *O*-allylic thiocarbamates. Intramolecular C(sp^3^)-arylation gave thiol derivatives **84** with retention of configuration.^63,64^ The synthetic utility of the thiols **85** (Scheme 25a) was demonstrated by ring-closing metathesis of **86**, giving enantioenriched dihydrothiophenes **87** bearing an α-quaternary stereocenter (Scheme 25b).^64^

## 5 Connective Routes to α-Tertiary Amines, Alcohols, and Thiols

The intramolecular S_N_Ar reactions of *N*-aryl ureas and (thio)carbamates described above all involve direct deprotonation to provide the required carbon nucleophile for the N → C aryl migration. An alternative connecting approach forms the α-carbanion by umpolung β-addition of a carbon nucleophile to α,β-unsaturated substrates, allowing an additional C–C bond to be formed in tandem with the T-S rearrangement.

**Scheme 26:**
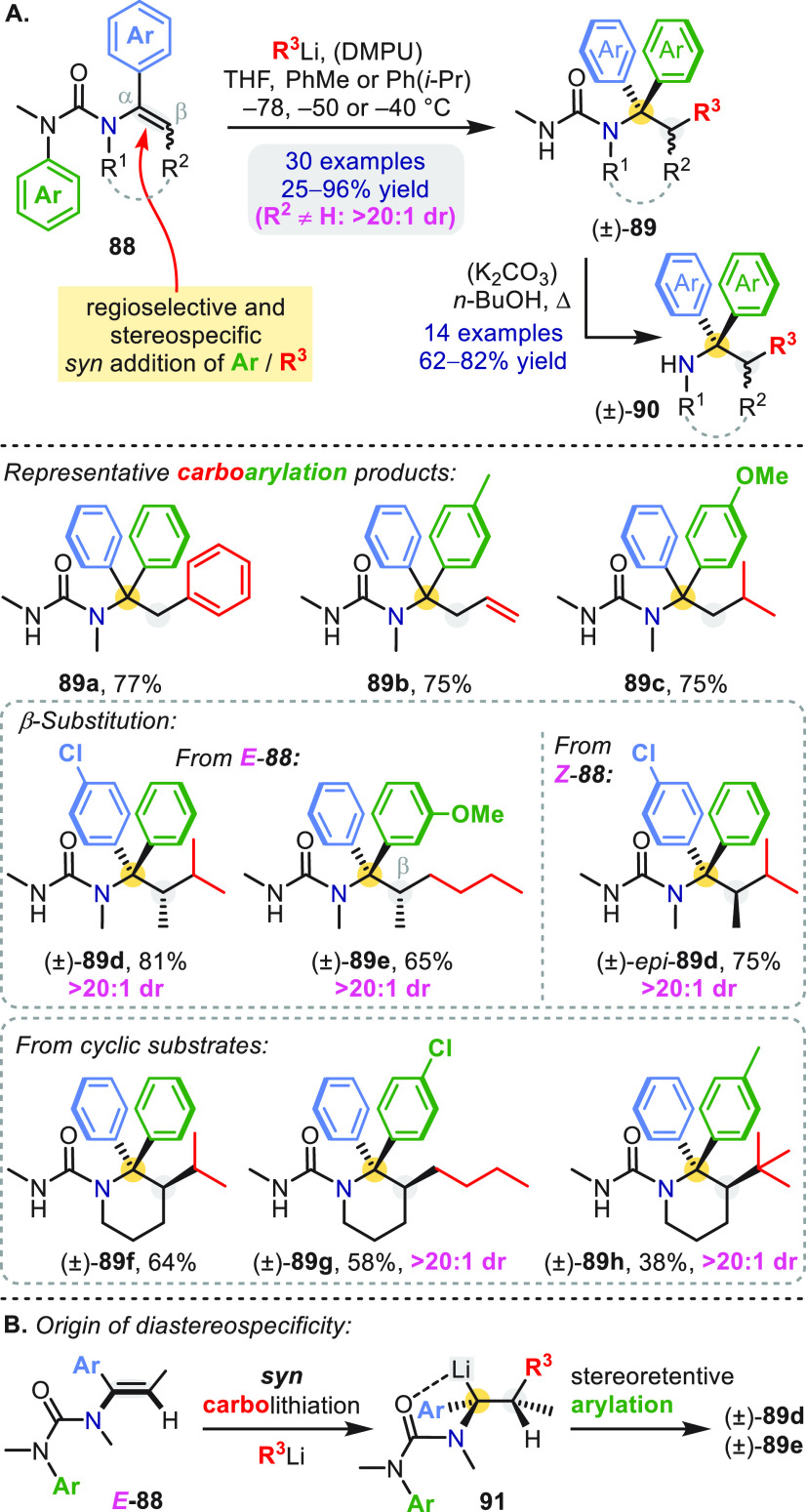
Carboarylation of *N*-Vinyl Ureas

With *N*-alkenyl ureas as starting materials (Scheme 26a), we found that an α-aryl substituent was needed to promote clean carbolithiation. Enamine derivatives **88** and an organolithium reagent gave the products **89** of successive “umpolung” β-addition and α-arylation.^65^ The process was completely regioselective and could be triggered by arylation, vinylation, or alkylation at the β-position (**89a**–**89c**). Reactions of geometrically defined substrates (R^2^ = Me) were diastereoselective: β-branched products (**89d** and **89e**) were formed with >20:1 dr from *E*-**88**, while exchanging *E*-**88** for the corresponding *Z*-isomer provided both diastereomers of a given product (**89d** and *epi***-89d**) with equally high levels of stereoenrichment; the fact that β-methyl, *Z*-vinyl ureas followed the carbolithiation/rearrangement pathway was notable in itself because of their known susceptibility to γ-deprotonation (Scheme 8).^42^ Cyclic substrates likewise reacted with complete stereospecificity (**89g**, **89h**).^46^

The relative stereochemistry of products arising from β-substituted substrates **88** (linear and cyclic) was consistent with *syn* carbolithiation followed by stereoretentive α-arylation (Scheme 26b).^46,65^ Free amines **90** were revealed by solvolysis in refluxing *n*-BuOH (Scheme 26a).^46,65^

**Scheme 27:**
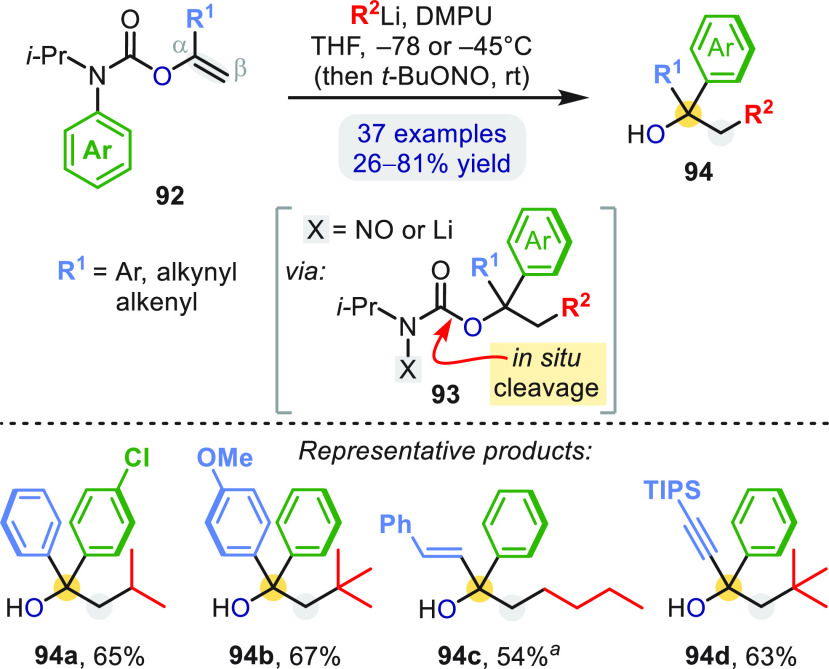
Carboarylation of *O*-Vinylcarbamates Using toluene as solvent and TMEDA as an additive.

Analogous β-alkylation/α-arylation was also possible with vinyl carbamates **92**, where an *N*-isopropyl group was needed to enforce chemoselective carbolithiation of the “enolate” alkene over direct attack at C=O (Scheme 27).^66^ The rearrangement to **93** (X = Li) was followed by *in situ* (CO)–O bond cleavage, either by reaction with the excess organolithium or, if required, by converting the remaining lithiated carbamate to the base-labile nitroso derivative (X = NO) before workup. This procedure conveniently afforded a range of tertiary alcohols **94**.

**Scheme 28:**
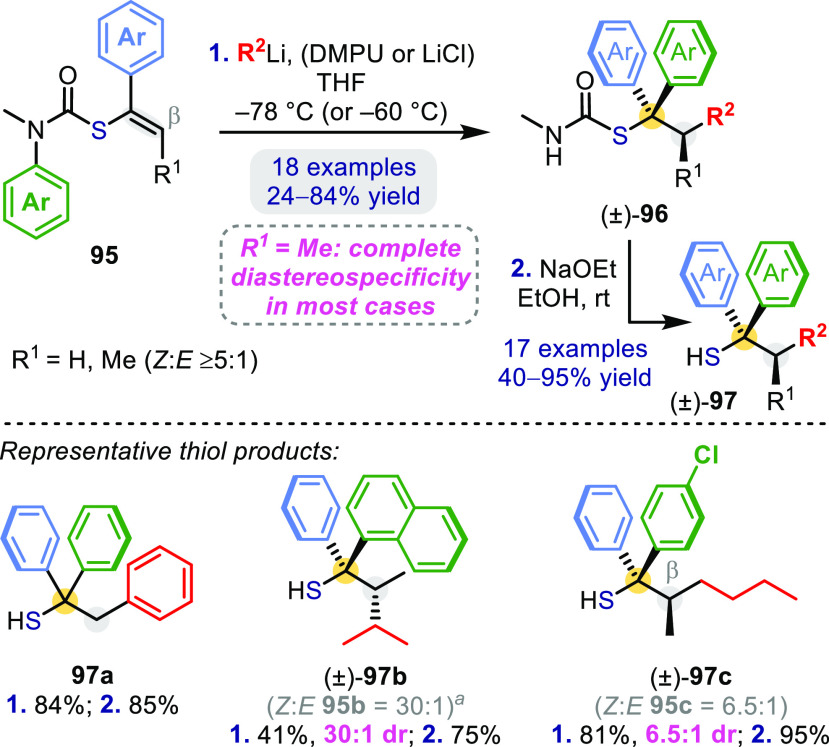
Carboarylation of *S*-Vinyl Thiocarbamates Starting material had an *N*-Et substituent.

This “umpolung” connective approach was further applied to *S*-vinyl thiocarbamates **95** (Scheme 28) to give a set of hindered tertiary thiols **97** carrying branched carbon chains:^67^ complete stereospecificity was observed in most cases.

**Scheme 29:**
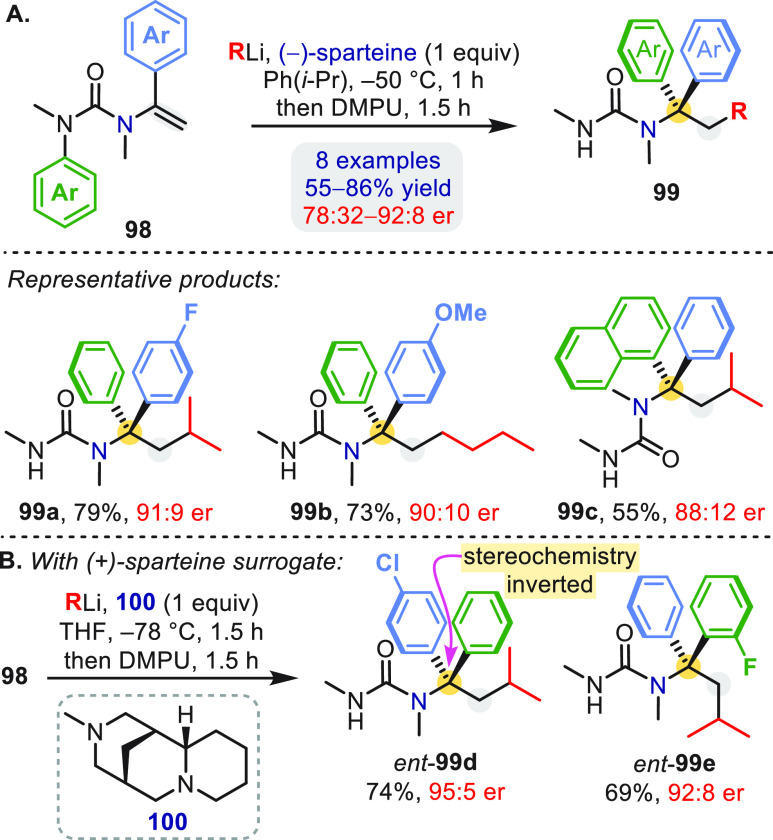
Enantioselective Carboarylation of *N*-Vinyl Ureas Configuration of **99** is corrected from original paper.^68b^

An asymmetric variant of the carboarylation of vinyl ureas was developed using (−)-sparteine as a ligand (Scheme 29a).^68^ The enhanced reactivity of the (−)-sparteine-complexed organolithium made possible a facially selective carbolithiation of **98**, producing an enantioenriched benzyllithium that, upon addition of DMPU, underwent enantiospecific T-S rearrangement^2^ to deliver **99**. The key to good enantioselectivity was the use of the noncoordinating solvent cumene, which allowed complete carbolithiation within 1 h at −50 °C and enhanced the configurational stability of the resultant organolithium. Products of opposite absolute configuration were obtained by exchanging the position of the aryl groups in **98** or, in some cases, by using the (+)-sparteine surrogate **100** as the chiral ligand in THF (Scheme 29b).^68^*O*-Vinyl carbamates **92** and *S*-vinyl thiocarbamates **95** gave enantioenriched products using (−)-sparteine or **100** only with modest enantioselectivity.^66,69^

**Scheme 30:**
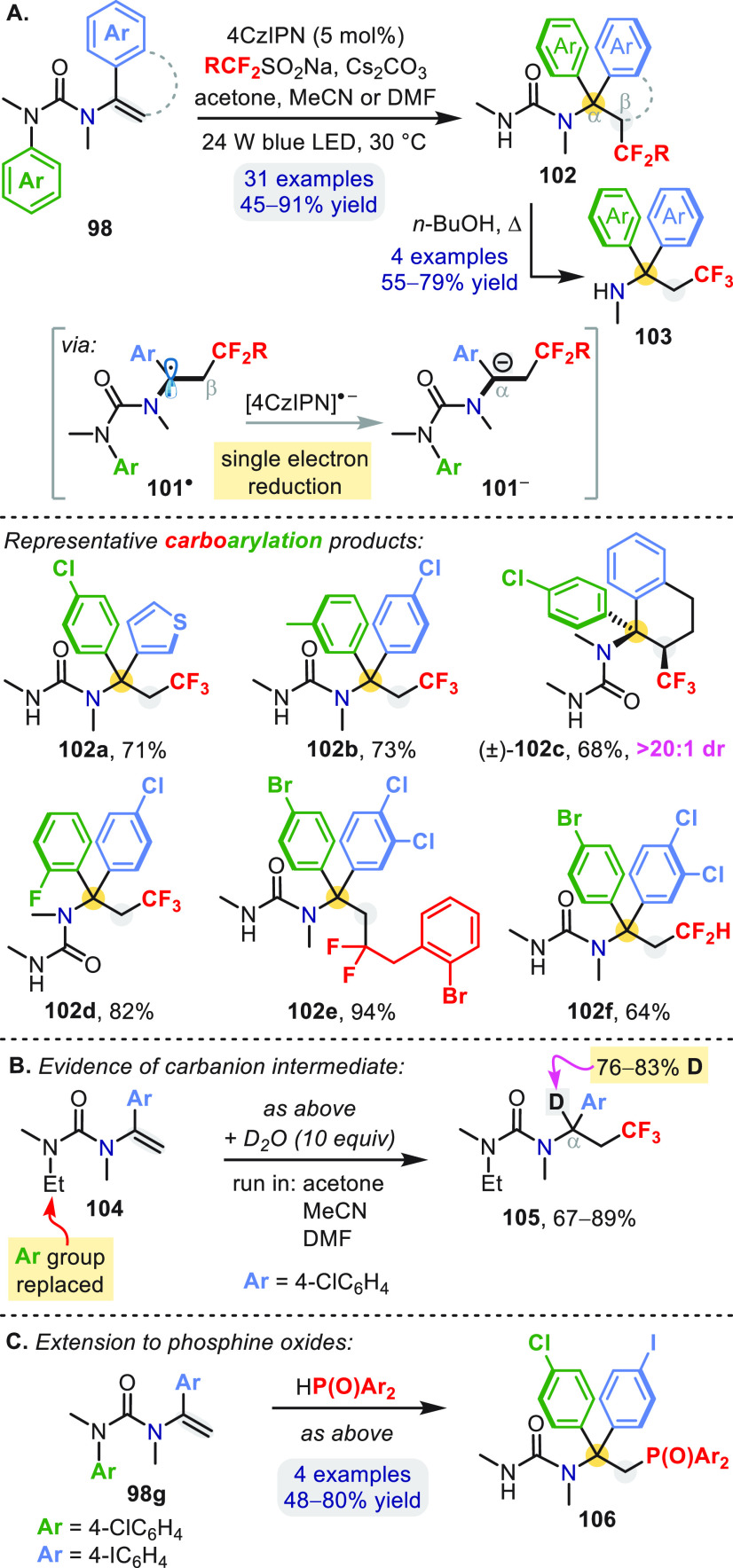
Photocatalytic Carboarylation of *N*-Vinyl Ureas

Carbolithiation has limited compatibility with reactive functional groups, but vinyl ureas **98** underwent more versatile photoredox-based carboarylation by way of the addition of carbon-centered radicals (Scheme 30a).^70^ A readily available organic dye (4CzIPn) was used to initiate a radical-polar crossover process, providing products **102** of tandem β-fluoroalkylation and α-arylation. The photoredox cycle involved oxidation of a sulfinate anion to release electrophilic fluoroalkyl radicals that underwent a polarity-matched β addition to **98**. Reduction of the resulting benzylic radical **101**^•^ by [4CzIPn]^•–^ closes the photoredox cycle and generates α-anion **101**^**–**^, which traps a variety of *N*′-aryl groups.

The standard potential of redox pair **101**^•^**/101**^**–**^ was challenging to measure directly, but compelling evidence of **101**^**–**^ as a viable intermediate was obtained by submitting modified *N*′,*N*′-dialkyl urea **104** to the standard conditions with D_2_O added as an anion quencher (Scheme 30b); significant α-deuteration (76–83%) of addition product **105** was observed in three different solvents with higher p*K*_a_ values and lower (C–H) bond dissociation energies than D_2_O.^70^ Furthermore, repeating the standard reaction of **98b** (to form **102b**) but with added D_2_O predominantly returned the carbodeuteration product (not shown), ruling out the possibility of a radical-based T-S rearrangement.^71^ Vinyl urea **98g** (Scheme 30c) also accepts P-centered radicals to give arylphosphonylation products **106** without any modifications to the standard conditions,^70^ suggesting that this photoredox approach holds wider promise for the construction of α,β-functionalized amines.

## 6 Conclusion and Outlook

The inherent conformational bias in acylated *N*-alkyl anilide congeners enforces spatial proximity between an *N*-aryl group and tethered carbanions and leads to remarkably versatile intramolecular S_N_Ar reactions. These stereocontrolled C-arylations transfer (hetero)aryl groups of diverse electronic and steric nature to an sp^3^ carbon without the use of transition metals. Our strategy not only enables unprecedented S_N_Ar reactivity for a wide range of nonstabilized and stabilized carbon nucleophiles but opens up access to rare or previously elusive compound classes such as tertiary thiols, α-aryl azepanes, and α-aryl quaternary amino acids.

Looking ahead, with conformational preorganization firmly established as a means of accelerating simple electrophilic arylation, we are seeking to apply the approach to the asymmetric arylation of enantiotopic secondary C–H bonds, and the catalytic, enantioselective arylation of stabilized carbanions. Amide tethers as conformational controllers^72,73^ also hold promise for the assembly of benzylic stereocenters.^74,75^ In addition, we are using the conformational preference of anilide congeners to develop other types of intramolecular transition metal-free couplings, including C-alkenylations.^76−79^

Our work demonstrates that the design of substrates where conformational bias predisposes intramolecular reactivity allows chemists to break free of the limitations of “classical” reactivity. Molecular conformation, whether by opportunity or design, is sure to continue to play a central role in the discovery of new ways to construct challenging C–C and C–X bonds.
